# The misplaced mouse Pax6 interneuron subclass: A cross-species transcriptomic reassignment

**DOI:** 10.1016/j.isci.2026.117632

**Published:** 2026-09-26

**Authors:** Jonathan M. Werner, Hamsini Suresh, Leon French, Jesse Gillis

**Affiliations:** 1 Physiology Department and Donnelly Centre for Cellular and Biomolecular Research, University of Toronto, Toronto, ON, Canada

**Keywords:** interneurons, cross-species cellular homology, single-cell transcriptomics, Patch-seq, spatial transcriptomics, cross-species neuroscience

## Abstract

Subclass cell types are strongly conserved across mammalian brains, making the systemic absence of a caudal ganglionic eminence (CGE) Pax6 interneuron subclass in mouse transcriptional atlases unexpected. Through cross-species transcriptomic analysis, we identify a Pax6 subclass homolog in mouse and uncover primate-specific divergence in the Sncg subclass. Analyses of electrophysiological and spatial datasets reveal limited subclass-specific structure in either modality, emphasizing the superior resolving power of transcriptomics for subclass distinctions. Our results highlight the pitfalls of relying on small sets of marker genes in cell-type annotation and provide evolution-aware annotations and marker sets to support robust cross-mammal interneuron comparisons.

## Highlights

• A transcriptional homolog of the primate Pax6 inhibitory subclass exists in mouse
• The Sncg inhibitory subclass may represent a primate-specific innovation
• Broad gene sets, rather than select markers, define cross-species subclass identity

## Graphical abstract

**Figure:**
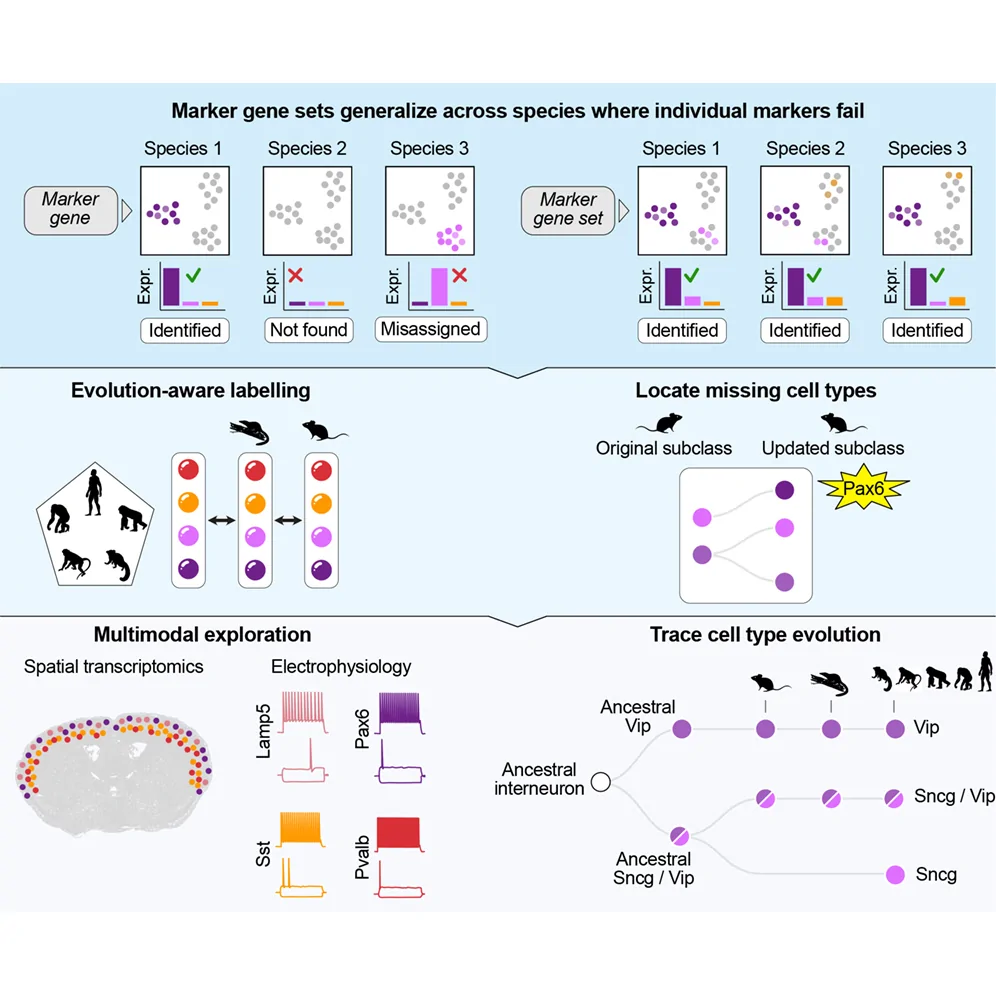

## Teaser

Neuroscience; Evolutionary biology; Transcriptomics

## Introduction

PAX6 is one of the most recognizable transcription factors in developmental biology.^1^ Originally identified as the causative gene underlying an eyeless mouse phenotype,^2^ subsequent studies have demonstrated strong conservation of PAX6 structure and function from insects to mammals across diverse developmental contexts.^2^^,^^3^^,^^4^^,^^5^^,^^6^ PAX6 plays a crucial role in neurodevelopment through its involvement in early cortical patterning^1^^,^^7^ and proper neurogenesis^1^^,^^8^; perhaps one of its more notable features is that *PAX6* is ubiquitously expressed in neural stem cells across developmental time points.^1^^,^^8^^,^^9^ Given this broad developmental influence, it is striking that adult primate single-cell brain atlases annotate a discrete caudal ganglionic eminence (CGE) interneuron subclass bearing its name.^10^^,^^11^^,^^12^^,^^13^^,^^14^^,^^15^ In contrast to the deeply conserved developmental biology of PAX6, mouse, the species with the deepest sampling and most refined transcriptomic taxonomies,^16^^,^^17^ routinely lacks any annotated CGE Pax6 interneuron subclass (Figure 1A).^16^^,^^17^^,^^18^^,^^19^^,^^20^^,^^21^^,^^22^

**Figure 1:**
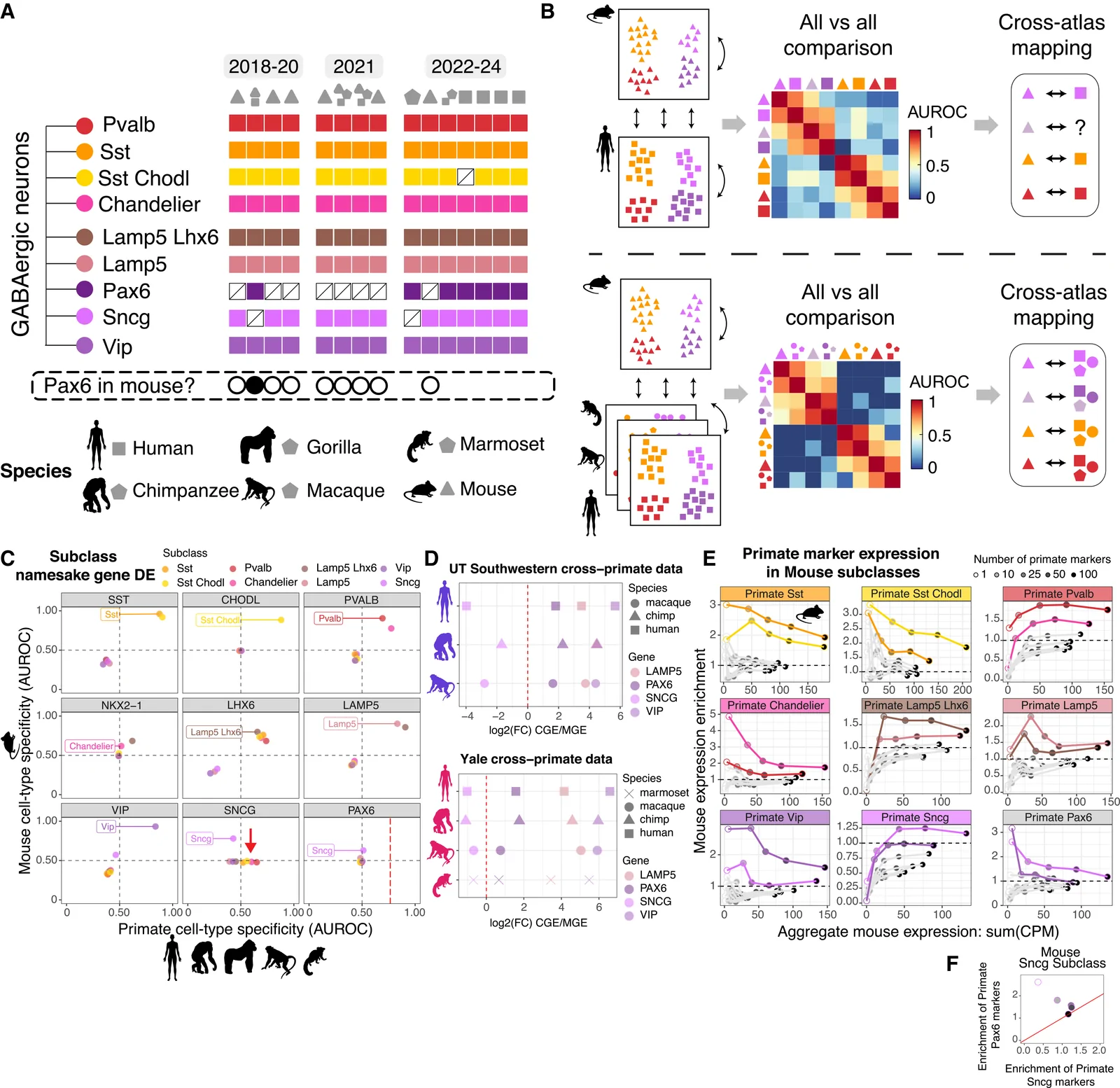
Cross-species differential expression comparisons suggest a Pax6-subclass misannotation in mouse datasets (A) Schematic depicting subclass-level annotations for mouse, non-human primate (NHP), and human single-cell datasets. Pax6 subclass annotations are consistently absent in mouse datasets, yet are consistently present in NHP and human datasets. (B) Schematic depicting how cross-species consensus cell-type annotations can help resolve cell-type alignment ambiguities when performing pairwise species comparisons. (C) Scatterplots comparing primate (Allen MTG dataset) and mouse differential expression (DE, MetaMarker AUROCs) for the namesake genes of the interneuron subclass annotations. The red arrow is highlighting the MGE-positive DE of the *SNCG* gene in primates. The red dotted line is depicting the primate *PAX6* AUROC in the primate Pax6 subclass, due to the absence of Pax6 subclass annotations in mouse. (D) Dot plots comparing the CGE/MGE log2 fold change of the *LAMP5*, *PAX6*, *SNCG*, and *VIP* genes across the primate species for the UT Southwestern and Yale datasets. Positive CGE DE is to the right of the dotted line. (E) Scatter-line plots comparing the aggregate mouse expression (summation of CPM values) against the mouse expression enrichment (see STAR Methods section) for increasing numbers of top primate subclass MetaMarkers (Table S1). Only the top 2 mouse subclasses per primate subclass are shown in color, determined by their maximal expression of the primate markers. (F) Scatterplot comparing the expression enrichment of the primate Pax6 and Sncg subclass markers for the mouse Sncg subclass.

On its surface, this may be a simple naming issue born out of the practice of using single marker genes as labels of cell types. Marker genes often drift across species,^23^^,^^24^^,^^25^ and *PAX6* expression alone need not define a conserved cell type. However, subclass-level identities are among the most stable and replicable elements of cortical organization: they persist across platforms, datasets, and species,^10^^,^^14^^,^^18^^,^^26^^,^^27^^,^^28^^,^^29^ even when finer clusters do not.^30^ The complete absence of a Pax6 subclass in mouse is therefore unexpected. Evolutionarily, a primate-specific Pax6 subclass would represent a significant and instructive divergence. Conversely, if the subclass exists in mouse but is misassigned, its absence exposes a failure point in cross-species cell-type annotation.

Prior work using early mouse neural taxonomies (e.g., Tasic et al.^22^) noted partial correspondences between human Pax6 interneurons and a Lamp5-annotated mouse cluster^31^^,^^32^ (Lamp5 Krt73), leaving open both possibilities: a primate expansion or a fragmented mouse homolog. Subsequent mouse atlases reported *PAX6* marker expression scattered across Lamp5, Vip, and Sncg subclass annotations,^16^^,^^19^^,^^20^^,^^21^^,^^22^ suggesting that any murine counterpart, if present, is dispersed by current labeling schemes. This ambiguity has consequences. Pax6-related phenotypes, whether developmental or functional, may already be present in mouse datasets but split across current subclasses, complicating comparisons within transcriptomic space and across other modalities that inherit these annotations.

The Pax6 inconsistency therefore offers a sharp test case for cross-species cell-type taxonomy. If a conserved Pax6 lineage exists, it should be recoverable by broad transcriptomic alignment. If not, the divergence indicates genuine cell-type evolution within CGE-derived interneurons toward a primate-specific innovation. Either outcome informs how we interpret subclass stability and how we evaluate cross-species cell-type correspondence more broadly.

To address the apparent case of the missing mouse Pax6 interneuron subclass, we performed a meta-analysis of CGE and MGE (medial ganglionic eminence) interneuron subclasses across diverse mouse and primate datasets (see STAR Methods). Our transcriptomic data collection includes mouse and primate (human, chimpanzee, gorilla, macaque, and marmoset) interneurons from both 10× and Smart-seq platforms, single-cell and single-nucleus preparations, and 18 mouse and 3 primate brain regions (Figure S1). Data were drawn from the Allen Institute (mouse and primate), Broad Institute (mouse), Yale (primate), and UT Southwestern (primate), enabling a broad evolutionary comparison to resolve transcriptomic cell-type annotation ambiguities (Figure 1B). Summarily, we used replicability-based cross-dataset alignment to re-evaluate transcriptomic subclass identity and then examined the resulting homologous groups across electrophysiological^20^ and spatial modalities.^16^ This approach clarifies whether the Pax6 subclass is conserved, misassigned, or truly primate specific and provides a framework for understanding how subclass-level identities persist or diverge across mammalian evolution.

## Results

### Marker aggregation strengthens cross-species differential expression signal

We started mouse and primate subclass comparisons by leveraging previously established cross-primate consensus interneuron subclasses from 5 primate species^14^^,^^27^ (Allen Institute data, Figures S2 and S3). This prior work identified nine conserved primate interneuron subclasses: Pvalb, Chandelier, Sst, Sst Chodl, Lamp5, Lamp5 Lhx6, Vip, Sncg, and Pax6. We then curated a high-confidence subset of mouse data by first identifying replicable cell clusters across our 8 diverse Allen and Broad Institute mouse datasets using MetaNeighbor,^33^^,^^34^ which utilizes cell-cell gene expression similarity to identify replicable cell types across datasets (Figure S4, see STAR Methods).

We initially compared differential expression (DE) of namesake marker genes for each subclass, including *PAX6*, to assess where *PAX6* marker expression aligns with current mouse subclass annotations (Figure 1C). Several markers show expected conserved DE across species (*PVALB*, *SST*, *CHODL*, *LAMP5*, *LHX6*, and *VIP*), but we also identify striking species-specific patterns for the Chandelier^35^ and Sncg subclass namesake genes, though most dramatically for *SNCG* (Figure 1C). *SNCG* is a strong marker for the mouse Sncg subclass but is unexpectedly positively DE in primate MGE-derived rather than CGE-derived subclasses. A strong transcriptomic separation of CGE- and MGE-derived subclasses is a consistent finding across studies,^10^^,^^11^^,^^14^^,^^19^^,^^20^^,^^21^^,^^22^^,^^26^^,^^27^ marking the apparent MGE switch in *SNCG* DE within primates as particularly surprising. We confirm the MGE-positive DE of *SNCG* in both the Yale and UT Southwestern primate datasets (Figures 1D and S5), validating a significant shift in DE patterns between mouse and primates. Importantly, we also observe mild positive DE of *PAX6* in the mouse Sncg subclass, suggesting misannotation rather than a true loss of Pax6-like interneurons in mouse. However, this assumes *PAX6* is a reliable cross-species subclass marker, which the cross-species variability of *SNCG* expression demonstrates is not guaranteed. To that point, the poor DE of *SNCG* within the primate Sncg subclass suggests that this subclass should be named differently for primates, but for the sake of interpretation with respect to existing annotations, we keep all currently published cell-type annotations when detailing results.

The CGE-to-MGE inversion of *SNCG* underscores how even well-established subclass markers can diverge across species, challenging the reliability of single or small marker sets for cross-species annotation. To test the robustness of subclass identity, we quantified the enrichment and aggregate expression of increasing numbers of primate subclass markers (Table S1) in mouse data. While top individual markers can show weak or inconsistent DE in the expected mouse subclasses (e.g., Pvalb, Lamp5, Lamp5 Lhx6, and Sncg; Figure 1E), even extending to sets of just ten markers in aggregate reliably recovers expected trends (Figure 1E). Notably, primate Pax6 markers are more enriched in the mouse Sncg subclass than primate Sncg markers, further supporting a misannotation across species (Figure 1F). These findings suggest that although individual marker genes may be unreliable, aggregate subclass profiles are conserved and provide a robust basis for cross-species alignment.

### A transcriptional Pax6 subclass homolog in mouse

We used a one-vs.-best MetaNeighbor analysis (see Data S1/Methods S1) to systematically map mouse interneuron clusters to primate subclasses based on transcriptomic replicability (Figures 2A and S6). This approach identifies the best and next-best primate subclass matches for each mouse cluster and assesses how well a mouse cluster distinguishes its best primate subclass relative to its next-best match, a stringent test of replicability across species. We identified robust 1-to-1 matches between mouse clusters and all nine primate subclasses, including Pax6 (Figure 2A). The transcriptional homologous mouse subclass matches are represented by all 8 mouse datasets and span 18 brain regions, demonstrating the strong consensus subclass alignment between primates and mice in light of significant technical and biological variability (Figures S7A and S7B).

**Figure 2:**
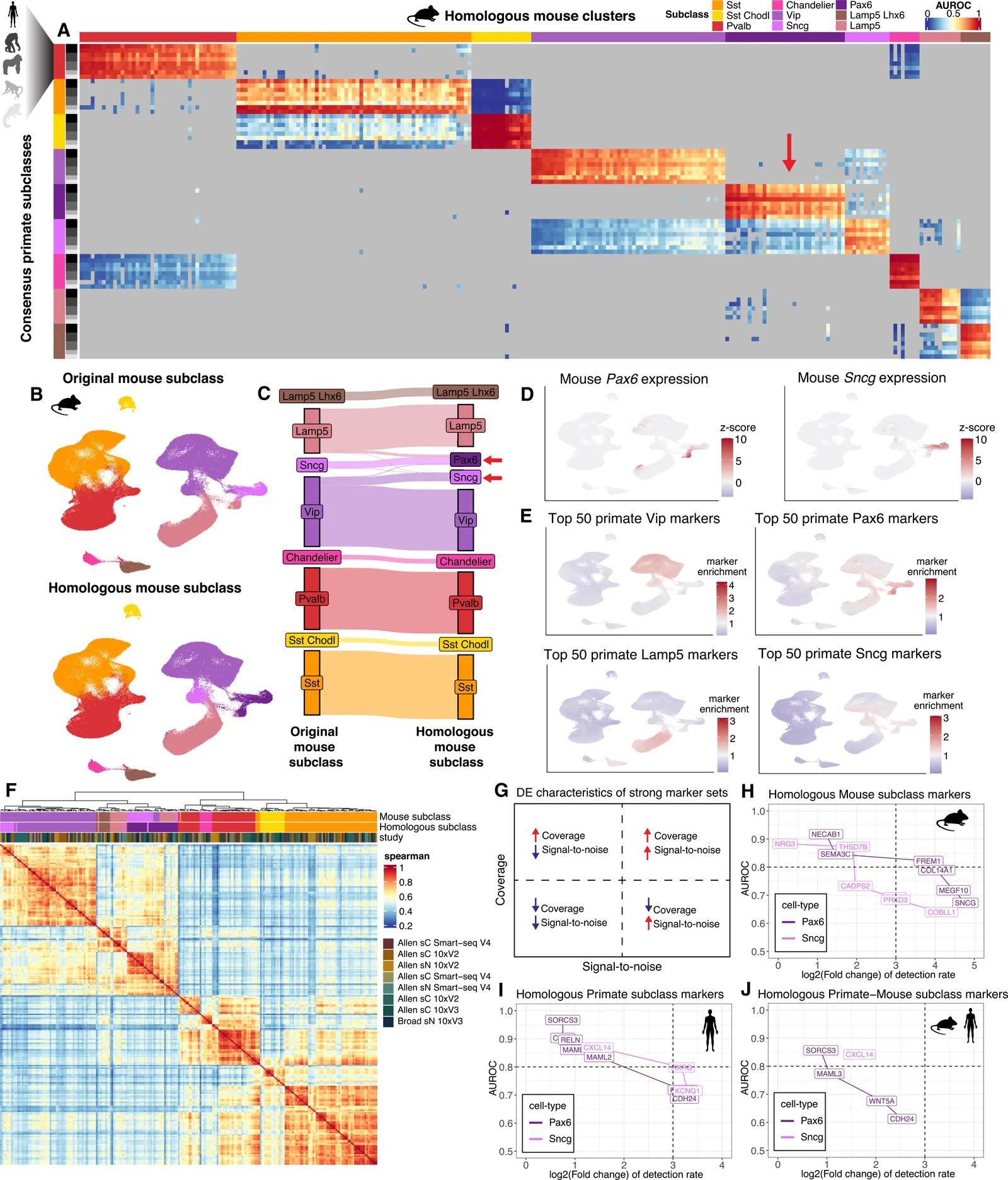
All 9 primate interneuron subclasses have transcriptional homologous matches in mouse (A) One-vs.-best MetaNeighbor scores for the replicable mouse clusters (columns) compared to the consensus primate subclasses (rows). Each mouse cluster receives an AUROC for only the top 2 subclass matches per primate dataset (16 total AUROCs for each column from the 8 total primate datasets, see Figure S1). The red arrow is highlighting the mouse clusters aligned to the primate Pax6 subclass. Gray squares indicate mouse-cluster/primate-subclass pairs that are not in the top 2 matches per dataset comparison. See Note S1 for more details. (B) Uniform manifold approximation and projections (UMAPs) depicting the integrated replicable mouse clusters with either their original (left) or homologous subclass annotations (right, from A). (C) Sankey plot depicting the contributions to the homologous mouse subclass labels from the original mouse subclass labels. The red arrows highlight the contributions resulting in the homologous Pax6 and Sncg subclass annotations. (D) Same UMAP as in (B), depicting the *Z*-scored expression of the mouse *PAX6* and *SNCG* gene expression. (E) Same UMAP as in (B), depicting the marker set enrichment scores (see STAR Methods section) for the top 50 primate Vip, Pax6, Lamp5, and Sncg markers (Table S1) in the mouse data. (F) Cell-type taxonomy (see STAR Methods section) of the integrated replicable mouse clusters with high primate homology. (G) Schematic outlining different categories of marker genes determined by coverage and signal-to-noise thresholds. Thresholds are derived from Fischer and Gillis.^29^ (H) Pareto front markers for the Pax6 and Sncg mouse homologous subclasses computed from the AUROC and log2 fold change of detection rate differential expression statistics, as proxies for coverage and signal-to-noise measurements. Sncg markers: *NRG3*, *THSD7B*, *CADPS2*, *STAC*, *PRKD3*, and *COBLL1*. Pax6 markers: *NECAB1*, *SEMA3C*, *FREM1*, *COL14A1*, *MEGF10*, and *SNCG*. (I) Same as in (H), but for homologous subclass markers computed using the Allen cross-primate data. Sncg markers: *CXCL14*, *RXRG*, and *KCNG1*. Pax6 markers: *SORCS3*, *CDH4*, *RELN*, *MAML3*, *MAML2*, *KCNG1*, and *CDH24*. (J) Same as in (H), but for homologous subclass markers computed using the 8 mouse and Allen cross-primate datasets. Sncg markers: *CXCL14*. Pax6 markers: *SORCS3*, *MAML3*, *WNT5A*, and *CDH24*.

To resolve the prior absence of Pax6 in mouse subclass annotations, we compared original mouse subclass labels to their primate-aligned identities (Figures 2B and 2C). Nearly all previously annotated mouse Sncg clusters align instead with the primate Pax6 subclass, alongside subsets of Lamp5 and Vip clusters. Conversely, the mouse clusters most similar to the primate Sncg subclass are largely annotated as mouse Vip clusters. Marker expression further clarifies this reclassification: the majority of mouse *Pax6* expression is localized to the Lamp5 cells remapped to primate Pax6, while mouse *Sncg* marks the Sncg-to-Pax6 clusters (Figure 2D). This suggests that the homologous primate Pax6 subclass in mice is largely composed of the previous mouse Sncg subclass, along with a *PAX6*-expressing previous Lamp5 subset. Expanding to the top 50 primate subclass markers (Table S1) shows clear enrichment of the primate Vip, Lamp5, and Pax6 markers in the corresponding remapped mouse populations, while primate Sncg markers surprisingly lack strong and specific expression in the mouse data (Figure 2E).

We explored the homologous subclass label changes further with both expression enrichment and all-vs.-all MetaNeighbor analysis, which confirms Pax6 as the top primate subclass match for the associated remapped mouse clusters (Figures S7C and S7E). In contrast, although primate Sncg remains the best overall match for its mapped mouse clusters (Figure S7E), the weak marker enrichment (Figure S7D) and small AUROC (area under the receiver operating characteristic statistic) differences from competing subclasses (Figure S7G) suggest poor replicability. These patterns suggest that while the Pax6 subclass is robustly transcriptionally conserved across species, Sncg is a weaker, potentially less reliable subclass annotation.

### Mouse transcriptional similarity of homologous subclass annotations

Transcriptional similarity between clusters forms the basis of modern cell-type taxonomies, where hierarchical structure reflects graded levels of shared expression variability across cell clusters. We quantified cluster-level transcriptional similarity within individual datasets (Figure S8) and in an integrated mouse interneuron dataset (Figure 2F) to compare the structure recovered by the original subclass annotations versus our homologous subclass labels. Under the homologous annotations, Pax6-annotated mouse clusters form a coherent and well-separated group that is transcriptionally distinct from other subclasses. Importantly, the Vip and Lamp5 clusters that remap to Pax6 are more similar to one another than to the remaining Vip and Lamp5 clusters, indicating that the homologous Pax6 subgroup captures shared transcriptional structure that was diffuse under the previous labeling.

Similarly, the homologous mouse Sncg subclass, comprising a subset of clusters previously labeled Vip, forms a distinct outgroup compared to other Vip clusters. We investigate the homologous Sncg subclass further in a later section. Altogether, these patterns show that the homologous subclass labels better reflect the intrinsic transcriptional structure of mouse interneuron populations.

### Cross-species marker gene characteristics

With a robust transcriptional homologous mapping in hand, we next sought to identify marker genes that more faithfully represent subclass identity across species. Marker genes can vary in statistical properties relevant for cell-type representation, for example, signal-to-noise (degree of enrichment relative to background) and coverage (fraction of cells expressing the marker). While ideal markers will score highly on both, genes high in only one property can still be informative, especially when aggregated (Figure 2G).

We computed species-specific and cross-mammal markers for each homologous subclass (Table S1). A useful visualization of marker genes is a Pareto front, which defines a set of optimal marker genes given the objectives of having both high signal-to-noise and coverage.^29^ Focusing on the subclasses with inconsistent cross-species annotations, Figures 2H–2J present the Pax6 and Sncg subclass marker Pareto fronts for mouse, primate, and primate-mouse computed markers (obscured gene names are provided in the figure legend). *FREM1* is a high-performing marker for the homologous mouse Pax6 subclass; this aligns with the recent neurodevelopmental analyses identifying *Frem1* as a marker of the mouse Sncg subclass (previous non-homologous label),^36^ a stable DE finding that spans developmental and adult time points. Consistently across primates and the primate-mouse Pax6 subclass markers, *SORCS3*, *MAML3*, and *CDH24* are Pareto front markers, indicating these genes as conserved Pax6 subclass markers. Notably, *CXCL14* is identified as the optimal Sncg subclass marker across mouse and primates, but *CXCL14* also has strong DE for the Pax6 subclass as well (Table S1). The lack of conserved markers that are specific to the Sncg subclass aligns with our results indicating a poor Sncg match between mouse and primates.

As expected, marker statistics generally decrease when computed over increasingly diverged species (Figure S9), and the primate-mouse Pareto front of markers for most of the subclasses excluding Pax6 and Sncg narrows to just the namesake marker genes (Figure S9; *SST*, *PVALB*, *LAMP5*, and *VIP*). Across all interneuron subclasses, only a limited number of genes per subclass maintain high DE statistics across the rodent and primate lineages (Figure S9). These results reinforce that single-gene markers can lack statistical discriminability across species, and cross-species subclass definitions are best supported by multigene transcriptional signatures.

### Electrophysiological variability of homologous mouse interneuron subclasses

Because transcriptional identity can reflect underlying regulatory programs shaping other modalities, we next examined how transcriptional homologous subclass labels capture variation in expression of genes directly tied to electrophysiological features. We began with a CGE-only (due to the prior cross-species annotation inconsistencies) transcriptomic dimension reduction using 176 ion-channel-related genes (Table S2). Even using this restricted gene set, homologous CGE subclasses remain separable, and DE analysis of ion channel genes using the original mouse subclass labels still yields patterns better aligned with our homologous annotation structure (Figures 3A, 3B, and S10). Thus, the improved coherence of homologous labels is robust to a constrained transcriptomic feature space tied to electrophysiological characteristics.

**Figure 3:**
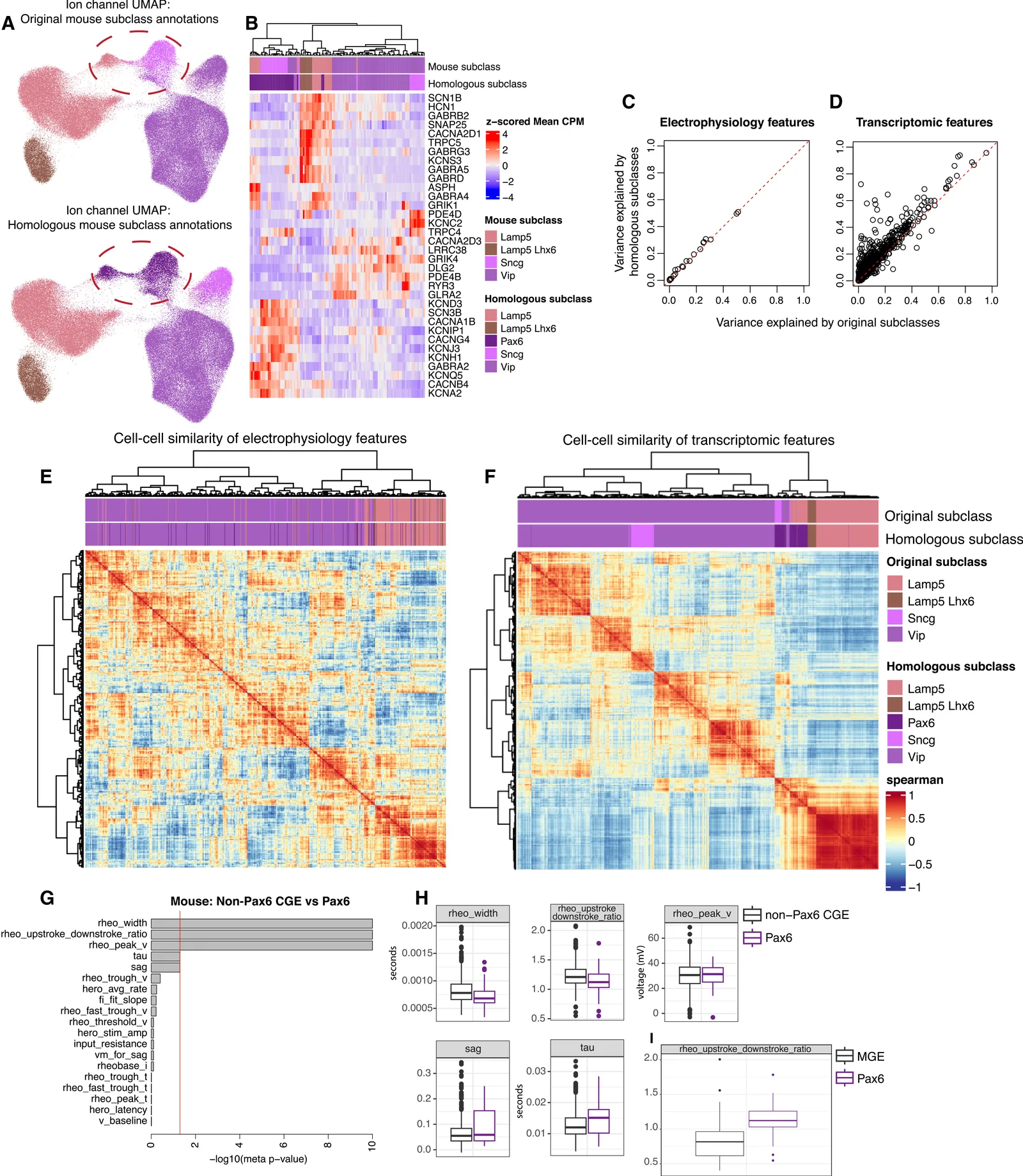
Electrophysiological variability of homologous mouse interneuron subclasses (A) UMAP of integrated homologous mouse CGE clusters using 176 ion channel genes (Table S2). (B) Heatmap of *Z*-scored mean CPM (counts per million) values for the top 10 differentially expressed ion channel genes computed using the original subclass labels. The *Z*-scored CPM values of all 176 genes are presented in Figure S10A. (C) Omega-squared values per electrophysiological feature using original subclass labels (*x* axis) compared to the homologous subclass labels (*y* axis). See STAR Methods section. (D) Omega-squared values per gene for the top 2,000 highly variable genes using original subclass labels (*x* axis) compared to the homologous subclass labels (*y* axis). See STAR Methods section. (E) Heatmap depicting the Spearman correlation coefficients between cells computed using *Z*-scored electrophysiological features. (F) Heatmap depicting the Spearman correlation coefficients between cells computed using *Z*-scored transcriptomic features. Only the top 25 highly variable genes were used to match the dimensionality of the electrophysiological data. (G) Bar plot depicting the aggregate *p* values per electrophysiological feature for distinguishing Pax6 cells from non-Pax6 CGE cells. See STAR Methods section. (H) Boxplots comparing the significant electrophysiological features identified in (G) between the Pax6 and non-Pax6 CGE cells. Additional details on these measurements can be found in the original publication.^20^ (I) Boxplot comparing the rheobase upstroke/downstroke ratio between MGE and Pax6 cells.

To quantify how well transcriptional homologous subclass labels explain variance in electrophysiology, we leveraged a large mouse Patch-seq dataset^20^ transcriptionally matched to one of our analyzed atlases.^22^ We quantified, via omega-squared ANOVA effect size, the variance explained in electrophysiological features by the original versus homologous subclass labels (Figure 3C). Omega-squared penalizes overfitting, ensuring that the increase from four to five CGE subclasses does not inflate explanatory power. The homologous labels explain slightly more electrophysiological variance, but the improvement is small, especially compared to gene expression, where homologous labels produce a near-global increase in explained variance (Figure 3D).

When computing cell-to-cell similarity from electrophysiological features alone, the dominant axis of variability corresponds to a Vip-Lamp5 continuum, with no clear organization of Pax6, Sncg, or Lamp5-Lhx6 cells (Figures 3E and S10B). In contrast, transcriptional similarity computed from only the top 25 highly variable genes (to mirror the dimensionality of the electrophysiology data) cleanly recovers all five homologous CGE subclasses (Figure 3F). This highlights the far higher resolving power of transcriptomic measurements relative to electrophysiology for subclass-level distinctions. It is important to note that this is not entirely surprising, as these subclass labels were originally defined using transcriptomic data. Additionally, the ion channel genes sampled in Figure 3B and the highly variable genes sampled in Figure 3F extend in function beyond the electrophysiological phenotypes sampled in the Patch-seq dataset, inherently providing a larger sample of biological variability for defining subclass identity.

### Subclass-specific electrophysiological features

While electrophysiological structure does not recapitulate all CGE subclasses globally, we asked whether the newly annotated Pax6 subclass shows features that separate it from all other CGE subclasses. We tested for electrophysiological features that significantly distinguish Pax6 cells in aggregate using the non-Pax6 CGE subclasses as the empirical null (Figures 3G and 3H). Additionally, we targeted subsets of Pax6 cells in relation to their previous subclass labels, specifically testing for electrophysiological features that distinguish Pax6 cells previously annotated as Lamp5 from other Lamp5 cells (Figures S10C and S10D) and Pax6 cells previously annotated as Vip from other Vip cells (Figures S10E and S10F). The small sample size (*n* = 2) of the Vip-Sncg cells, the other major transcriptional homologous subclass reassignment, precludes robust investigations into their electrophysiological properties.

Across all these comparisons, the consistent electrophysiological features that distinguish Pax6 cells from all other CGE subclasses are related to differences in repolarization timing, specifically lower rheobase spike width and rheobase upstroke-downstroke ratio (Figures 3G, 3H, and S10C–S10F), indicating a slightly faster firing pattern in Pax6 cells. Additionally, the rheobase upstroke-downstroke ratio was the only feature that significantly distinguished Pax6 cells from MGE subclasses (Figure 3I), with MGE cells exhibiting lower ratios and thus a faster firing pattern, consistent with prior observations that MGE cells typically exhibit faster spiking patterns than CGE cells.^20^ Overall, although transcriptionally robust and distinct, the CGE subclasses are not comparably separable electrophysiologically, and the dominant axis of electrophysiological variation is defined across Vip and Lamp5 cells. While this may be attributed to a sampling size issue, the Patch-seq dataset we employ is notably the largest of its kind, highlighting a limitation in currently available data.

### Spatial variability of homologous mouse interneuron subclasses

Spatially resolved transcriptomics provides yet another dimension for exploring subclass identity. Using a whole-brain mouse atlas from the Allen Institute,^16^ we computed the local composition of each cell’s nearest 100 neighbors to assess whether homologous subclasses occupy characteristic spatial niches. Beginning with a broad spatial resolution at the level of subclasses, spatial neighborhood composition does not show strong subclass specificity (Figure S11). Instead, MGE subclasses preferentially localize with deep-layer cell types, CGE subclasses with superficial-layer cell types, and both lineages show clusters localized with hippocampal cell types (Figure S11).

Increasing the cluster resolution to supertypes (as defined in Yao et al.^16^) and focusing on local spatial composition of either excitatory supertypes (Figure 4A, rows) or inhibitory supertypes (Figure 4B, rows) with our subset of inhibitory supertypes included in the cross-species alignment (Figures 4A and 4B, columns) essentially recapitulates the same trends as when using broader cell-type labels (Figure S11). CGE cell types tend to colocalize with superficial excitatory types and other CGE cell types (Figure 4B), while MGE cell types tend to colocalize with deep-layer excitatory cell types and other MGE cell types (Figure 4B).

**Figure 4:**
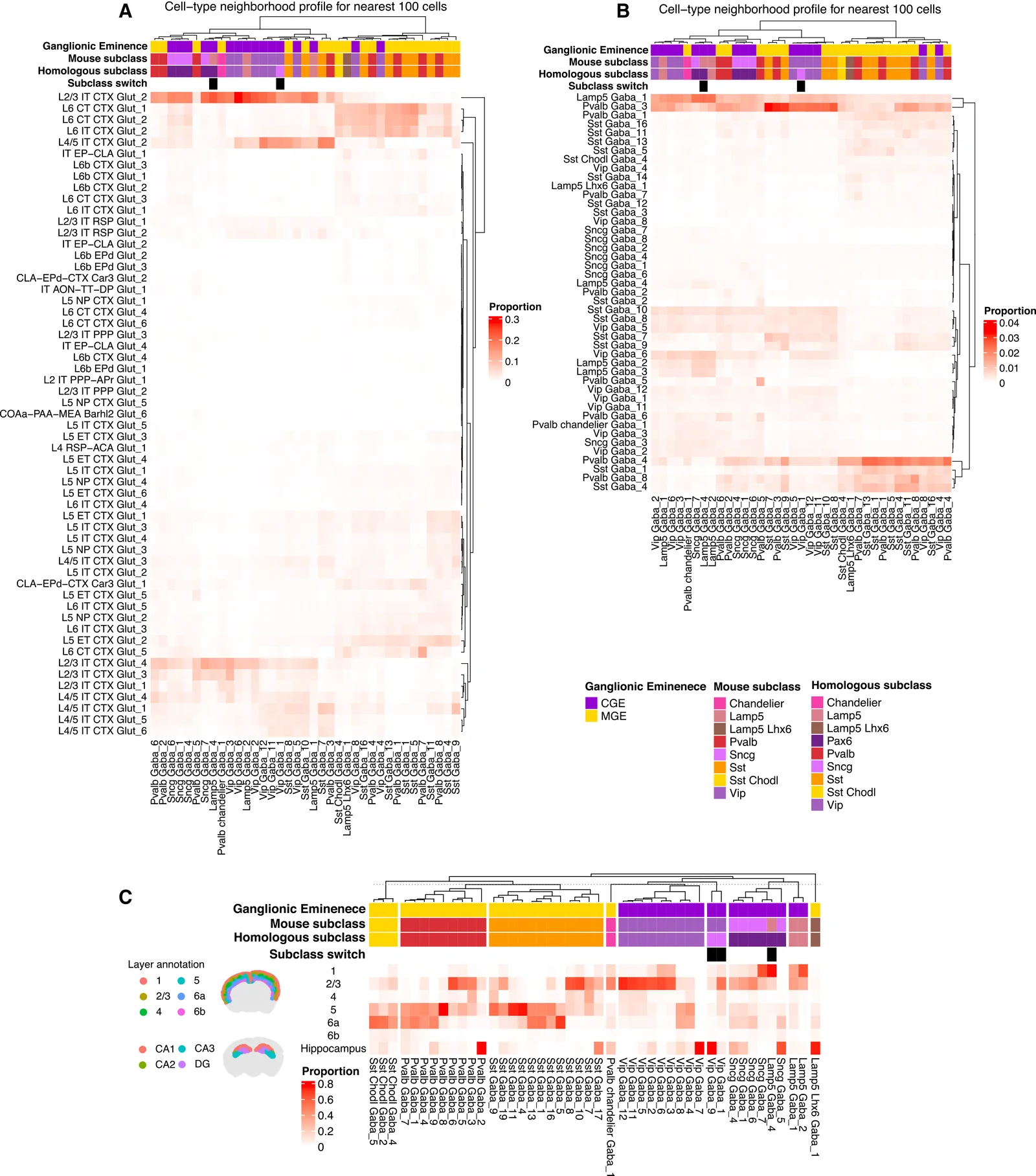
Spatial variability of homologous mouse interneuron subclasses (A) Heatmap depicting the average excitatory cell-type neighborhood proportion values for target MGE and CGE clusters (columns), computed using the top 100 nearest neighbors per cell. Columns and rows are at the lowest cluster level of the cell-type hierarchy presented in Yao et al.^16^ (B) Heatmap depicting the average inhibitory cell-type neighborhood proportion values for target MGE and CGE clusters (columns), computed using the top 100 nearest neighbors per cell. Columns and rows are at the lowest cluster level of the cell-type hierarchy presented in Yao et al.^16^ (C) Heatmap depicting the layer proportions of target MGE and CGE clusters (columns), using the layer annotations provided in Yao et al.^16^ Columns are grouped by subclass.

Quantifying layer localization in the cortex and hippocampus (Figure 4C) again reproduces these trends: predominant deep-layer localization for MGE clusters and superficial-layer localization for CGE clusters (Figure 4C), consistent with prior observations.^37^^,^^38^^,^^39^^,^^40^^,^^41^ However, most subclasses show exceptions to these layer preferences, for example, deep-layer Vip and superficial Sst clusters. A consistent spatial feature across subclasses, with the exception of Lamp5 cells, is the presence of both predominantly cortical and predominantly hippocampal clusters, which include the homologous Pax6 and Sncg subclass groupings (Figure 4C). In summary, while electrophysiological and spatial modalities provide valuable contextual information, neither modality cleanly recapitulates the transcriptionally defined subclass structure.

### Primate-specific divergence of the Sncg interneuron subclass

Our cross-species transcriptional mapping shows that, unlike Pax6, the Sncg subclass exhibits weak homology between mouse and primates, suggesting that it may represent a less robust subclass across mammals. To further assess the replicability of the Sncg subclass, we analyzed two additional primate datasets from Yale and UT Southwestern (Figures 5A, 5E, and S5). After *de novo* clustering, we predicted subclass identity of individual cells using the top 50 consensus primate subclass markers (Allen data, see STAR Methods). This approach yields clear 1-to-1 mappings of *de novo* clusters to all nine primate subclasses in both datasets (Figures 5B and 5F), confirming that the Sncg subclass is in fact highly replicable across primate datasets. This suggests that the poor match for the Sncg subclass in mouse reflects cross-species divergence, not instability within primates.

**Figure 5:**
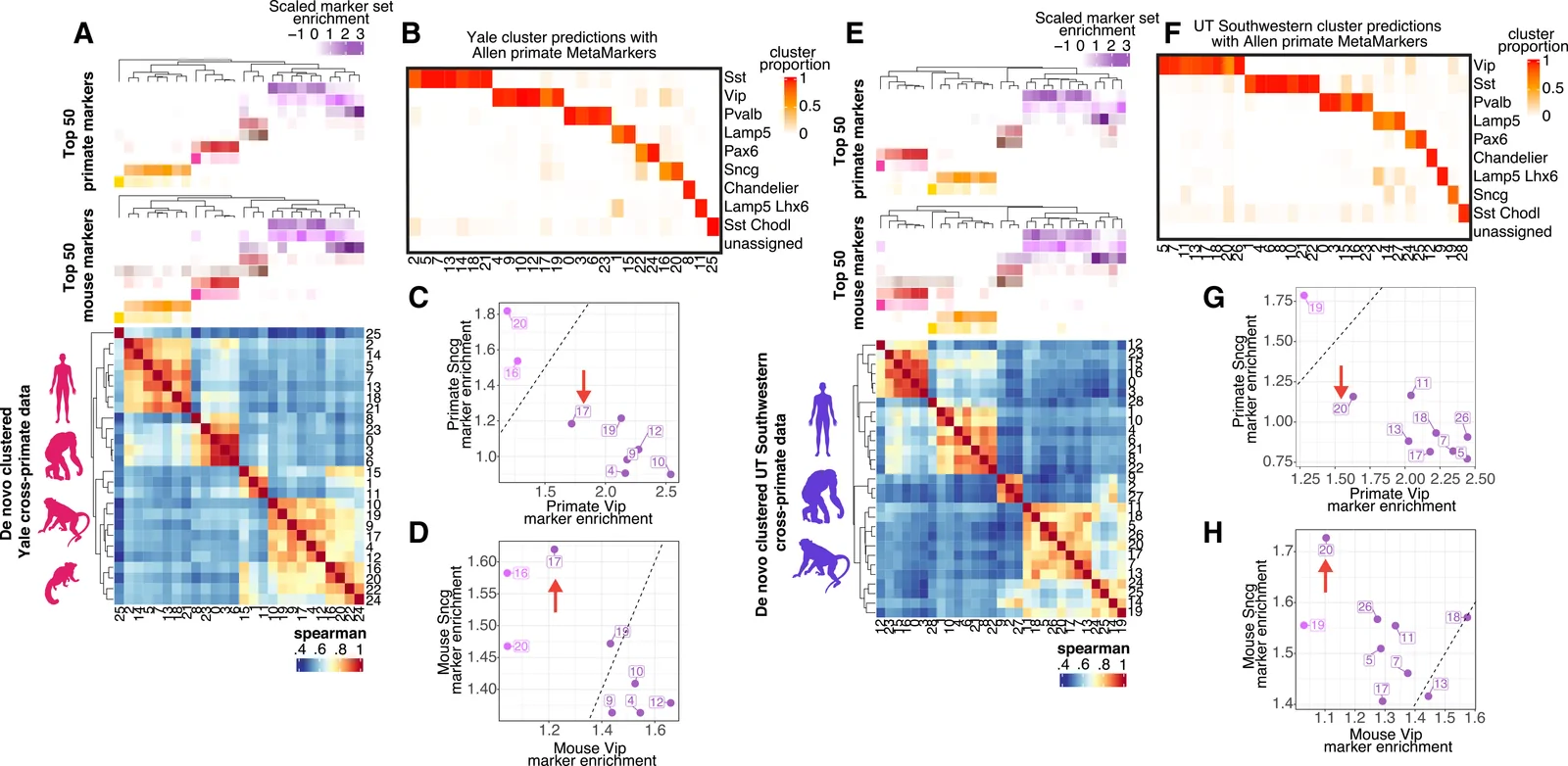
The homologous mouse Sncg subclass matches a primate Vip-Sncg hybrid cell type (A) Cluster taxonomy (see STAR Methods section) for the *de novo* Yale primate clusters. The heatmap depicts the Spearman correlations between cluster gene expression centroids for the cluster DE genes. The column annotations depict the *Z*-scored marker set enrichment scores for either the top 50 mouse or primate subclass markers. The color scales depict all positive *Z* score values. (B) Heatmap depicting the Yale *de novo* cluster proportions mapped to the Allen MTG primate subclass annotations using the top 50 primate subclass MetaMarkers. (C) Scatterplot comparing the primate Vip vs. primate Sncg marker set enrichment scores for the Yale *de novo* clusters that map to the primate Vip and Sncg subclasses (from B). The red arrow highlights the Yale Vip cluster (17) with the lowest Vip marker enrichment score. (D) Scatterplot comparing the mouse homologous Vip vs. homologous Sncg marker set enrichment scores for the same Yale *de novo* clusters as in (C). The red arrow highlights the same Yale Vip cluster 17 as in (C), which has the highest enrichment for the mouse Sncg marker set. (E) Same as in (A), but depicting the UT Southwestern cross-primate dataset. (F) Same as in (B), but depicting the UT Southwestern cross-primate dataset. (G) Same as in (C), but depicting the UT Southwestern cross-primate dataset. (H) Same as in (D), but depicting the UT Southwestern cross-primate dataset.

We next assessed how homologous mouse Sncg markers relate to primate subclasses. In both Yale and UT Southwestern datasets, these mouse markers exhibit intermediate enrichment in primate Sncg and Vip clusters, with the strongest enrichment occurring in a Vip cluster that expresses primate Vip markers only weakly (Figures 5C, 5D, and 5G–5H). This pattern matches our earlier observation that the homologous mouse Sncg subclass is essentially a Vip outgroup (Figure S8). Together, these results indicate that the primate Sncg subclass does not have a direct 1-to-1 transcriptional homolog in mouse; rather, its closest mouse analog is a Vip-adjacent outgroup.

To determine whether this divergence extends beyond mouse, we analyzed single-cell data from tree shrew,^42^ an evolutionary intermediate between rodents and primates (Figure S12). After identifying MGE and CGE homologous cells (see STAR Methods), we assessed enrichment for primate Vip, Pax6, Sncg, and homologous mouse Sncg markers (Figure 6A). Tree-shrew interneurons show clear enrichment for primate Vip and Pax6, but only mouse, not primate, Sncg markers are enriched. This again suggests that the primate Sncg subclass is a primate-specific innovation rather than a conserved mammalian subtype.

**Figure 6:**
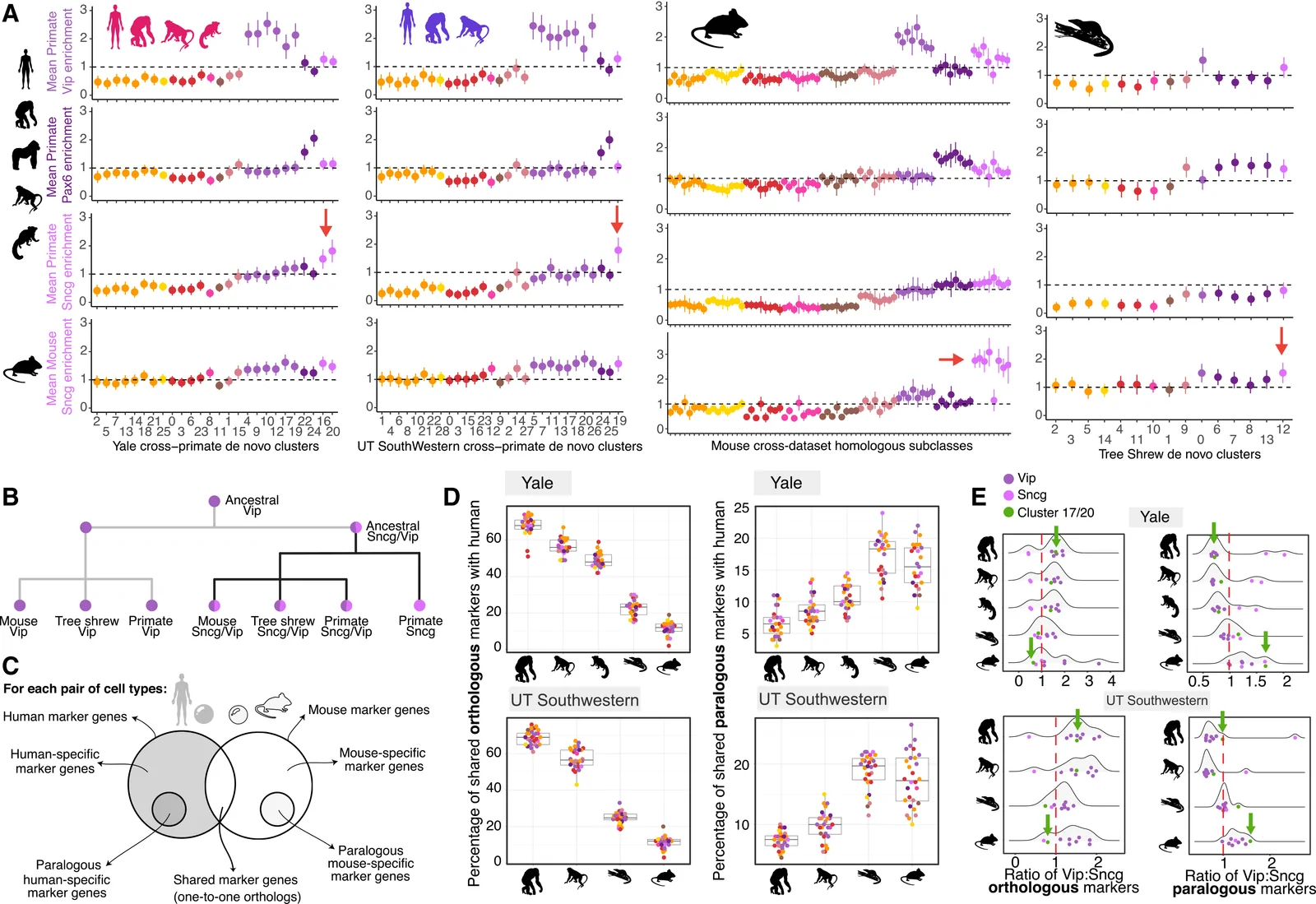
The Sncg subclass as a putative primate-specific innovation (A) Dot plots depicting the mean (±SD) marker set enrichment for the top 50 primate Vip, Pax6, Sncg, and the mouse homologous Sncg subclass markers (top to bottom) across the Yale, UT Southwestern, mouse, and tree-shrew datasets (left to right). The Yale and UT Southwestern *de novo* cluster subclass annotations are derived from Figures 5B and 5F. The mouse cluster subclass annotations are derived from Figure 2A. The tree-shrew *de novo* cluster subclass annotations are derived from mouse and primate marker set enrichment and MetaNeighbor assessments in Figure S12. The red arrows highlight the presence of clearly enriched primate Sncg subclass markers in the two non-Allen primate datasets, with a lack of strong primate Sncg subclass marker enrichment in the mouse and tree-shrew datasets. (B) Model for Sncg subclass evolution. An ancestral Vip outgroup (the ancestral Sncg/Vip) is extant in mouse, tree shrew, and primates as the Vip outgroup/Sncg-like populations, with the Sncg subclass as a further diverged group within the primate lineage. (C) Schematic outlining the orthologous (shared) and paralogous definitions for cross-species comparisons. (D) Boxplots depicting the percentage distributions of either shared orthologous (left) or paralogs (right) between humans and non-human species for the Yale (top) and UT Southwestern (bottom) datasets. (E) Density and scatterplots depicting the ratios of either Vip:Sncg orthologous markers (left) or Vip:Sncg paralogous markers (right) for the Yale (top) and UT Southwestern (bottom) datasets. The green arrows highlight either the Yale cluster 17 or the UT Southwestern cluster 20.

We extended this comparison to rat cortex,^43^^,^^44^ identifying homologous subclass matches (Figure S13). As in mouse, the Sncg homolog in rat corresponds to a Vip outgroup, though with more variability across all cell clusters/subclasses, likely reflecting reduced sample sizes. Across rodents, the consistent recovery of a Vip-outgroup-like Sncg subclass reinforces that this relationship is rodent wide, not mouse specific.

As a brief summary, the best homologous match for the primate Sncg subclass appears to be a Vip outgroup cluster in mouse that is also retained in tree shrew. Based on shared marker expression (Figures 5C, 5D, 5G, and 5H) and broader transcriptomic replicability (Figure S5), a specific primate Vip population (Yale 17 and UT Southwestern 20) aligns with this non-primate Vip outgroup/Sncg-like population. A putative explanation is that the primate Sncg subclass evolved from a Vip outgroup precursor that is retained across species, as the homologous Sncg subclass in mouse and tree shrew and as a Vip outgroup in primates (Figure 6B).

To investigate this evolutionary trajectory, we quantified the proportion of shared orthologous versus paralogous markers across species, similar to Shafer et al.^25^ (Figure 6C). As expected, we observed a progressive shift from shared orthologs to shared paralogs with increasing evolutionary distance (Figure 6D), consistent with paralog sub-functionalization contributing to cell-type divergence.^25^ The human Vip outgroup clusters (Yale 17 and UT Southwestern 20) switch from expressing more Vip orthologs in non-human primates to more Sncg orthologs in non-primates (Figure 6E), the only *de novo* clusters to do so, reinforcing their alignment with the non-primate Sncg subclass. Concurrently, human Vip and Sncg clusters show increasing convergence on Vip paralogs, rather than Sncg paralogs, when compared to mouse (Figure 6E), and this is specific to Vip compared to other interneuron subclasses (Figure S12L). These data suggest the Vip and Sncg subclasses are converging transcriptionally in the transition from primates to rodents, concordant with the model that the primate Sncg subclass may be a remodeled derivative of an ancestral Vip lineage (Figure 6B). Assessments of species further diverged from primates will be necessary to confirm.

## Discussion

Our study revisits the transcriptional organization of CGE interneuron subclasses across mammals and highlights the importance of assessing cell-type identity using broad transcriptional programs rather than reliance on small marker sets. Using cross-species meta-analysis, we resolve a long-standing inconsistency in current mammalian interneuron annotations by identifying a robust transcriptional homolog of the primate Pax6 subclass that had been split across Vip, Lamp5, and Sncg mouse labels. Where the prior absence of a Pax6 subclass annotation in mouse suggested the Pax6 subclass may be a primate-specific innovation, we clarify that this is not the case; instead, the Sncg subclass exhibits stronger evidence of being an evolutionary novel subclass in primates. We show that while the Sncg subclass is highly reproducible across primates, its best homologous match in mouse, rat, and tree shrew is a Vip outgroup, suggesting that the primate Sncg population represents a lineage-specific elaboration of an ancestral Vip-like lineage. Importantly, we demonstrate that the *SNCG* gene exhibits striking mouse-specific patterns of DE and is not a strong marker for the homologous Sncg subclass. While we can suggest a subclass annotation that better reflects conserved marker gene expression (*RXRG* as the optimal cross-primate marker), a broader community-driven investigation incorporating other modalities, morphological data as an example, may lead to a more coherent annotation.

A central theme emerging from our transcriptomic analyses is that cellular identity is distributed across the transcriptome.^29^^,^^33^^,^^45^ While small sets of marker genes can work well within species, they can degrade rapidly with evolutionary distance.^14^^,^^26^ Our quantitative marker analyses show that very few genes, and often none, display both strong signal-to-noise and broad coverage across multiple mammalian species. This suggests that individual genes or even small gene modules can diverge and change specificity without disrupting the broader transcriptional program that maintains cell-type identity. Because of this, aggregate transcriptional similarity is a reliable basis for inferring transcriptional homology across species. Thus, an approach that emphasizes broad profiles first and then interprets species-specific deviations second provides a more stable foundation for defining and comparing cell types. Our homologous mapping of Pax6 and Sncg subclasses exemplifies this principle: small marker sets obscured these relationships, whereas broader transcriptome alignment revealed consistent evolutionary structure. As a practical recommendation, simply including broader single-cell cell-type DE results in publications beyond a few select marker genes would enable the community to engage with and test the replicability of broader transcriptional cell-type profiles.

Our analyses also address the common, yet unresolved, question of what best constitutes validation of a transcriptomic cell type?^46^^,^^47^ Uncertainty around transcriptomic cell-type definitions often centers on the concern that clustering algorithms may overpartition continuous variation.^48^ However, the best source of external validation is unclear; should every true transcriptomic type display a distinct spatial pattern, a characteristic electrophysiological profile, or a unique developmental origin? The answer is currently unknowable because we lack the comprehensive multimodal datasets required to infer the rules linking transcriptomic identity to other cellular modalities. In both the electrophysiological and spatial datasets, we observe an inability to delineate CGE subclasses that are cleanly separable transcriptomically. Specifically, electrophysiological similarity collapses largely onto a Vip-Lamp5 axis, and spatial distributions largely reflect deep- versus superficial-layer biases shared across many subclasses rather than subclass-specific signatures. One explanation for this discordance may simply be that the resolution and genomic coverage of current electrophysiological and spatial atlases are insufficient to resolve fine-grained transcriptomic populations.

Given these realities, we argue that internal transcriptomic consistency, i.e., replicability across technologies, datasets, brain regions, and, perhaps most importantly when available, species, should be the first benchmark for recognizing a transcriptional cell type. Distinct and evolutionarily persistent transcriptomic entities provide the most reliable substrate for asking whether, and how, other modalities reinforce or differentiate them. If a transcriptional population is conserved across species separated by millions of years, then that population has been maintained by stabilizing forces. Such cross-species persistence elevates a transcriptional cluster from a statistical construct to an evolutionary unit, one that has endured long enough to be shaped by natural selection.^47^^,^^49^^,^^50^ Similarly, when cross-species data are unavailable, transcriptional consistency across technical and/or biological batches are powerful benchmarks for resolving robust transcriptional cell types.

Importantly, our emphasis on transcriptomic conservation should not be interpreted as a claim that transcriptomes represent the definitive substrate of cell identity. Rather, transcriptomics is currently the modality that provides the greatest resolution and scalability for interrogating cellular diversity. The broader principle is that cell-type definitions should be grounded in the features that most reliably identify stable biological entities. Today, transcriptomics occupies that role. In the future, advances in single-cell proteomics may permit equally comprehensive comparisons of cellular identity across species. Because proteins are more directly coupled to cellular function than transcripts, proteomic conservation may ultimately provide an even stronger benchmark for evaluating cell-type homology. We therefore view transcriptomic taxonomy not as an endpoint but as a current best approximation of a deeper biological organization that future molecular technologies will continue to refine.

For future work regardless of modality, beginning with conserved units would provide a stable cross-species scaffold on which species-specific specializations can be layered. This evolutionary perspective will help distinguish broadly shared features of neuronal organization from lineage-specific innovations and provide a biologically grounded path forward as cell-type taxonomies continue to expand in scale and sampled modalities.

### Limitations of the study

The cross-primate consensus MTG (middle temporal gyrus) taxonomy serves as the backbone for our mouse-primate comparisons; consequently, the resolution of the homologous matches we identify across species is constrained by the structure and granularity of this taxonomy. Although our study focuses on transcriptionally replicable clusters across species, our inferences are inherently limited by the scope of the reference data available. In both the primate reference datasets and the mouse datasets, we exclude clusters that are not shared across all primate species, as well as mouse clusters that represent intermediate matches between primate subclasses (Table S3; Figure S6). Collectively, these excluded clusters comprise cellular populations that fall outside the strict subclass delineations used in our analyses. Such intermediate cases may reflect unresolved biological granularity across datasets and could represent genuine evolutionary intermediates that we currently do not characterize. As an additional limitation, we did not model or control for biological sex in our analyses, as in most cases this information was not included in the available sample metadata.

## Resource availability

### Lead contact

Requests for further information and resources should be directed to and will be fulfilled by the lead contact, Jesse Gillis (jesse.gillis@utoronto.ca).

### Materials availability

This study did not generate new unique reagents.

### Data and code availability

• This paper analyzes existing, publicly available data, accessible at the following sources: Tasic et al.^22^ (GEO: GSE111157); Yao et al.^16^ (10*×* RNA-seq data-GEO: GSE246717; MERFISH data: https://doi.org/10.35077/g.610); Yao et al.^18^ (NeMO: nemo:dat-ch1nqb7); Langlieb et al.^17^ (raw sequencing reads-NeMO: nemo:dat-aa0jwmj; processed data-https://www.braincelldata.org/); Jorstad et al.^14^ (raw sequencing reads-NeMO: nemo:dat-net1412; processed data:https://data.nemoarchive.org/publication_release/Great_Ape_MTG_Analysis/); Ma et al.^51^ (NeMO: nemo:dat-zkgd0it); Caglayan et al.^52^ (GEO: GSE192774); Xiong et al.^42^ (GEO: GSE229035); Gungor Aydin et al.^43^ (GEO: GSE262970); Wang et al. (GEO: GSE253672); and Gouwens et al.^20^^,^^53^ (raw electrophysiology data-DANDI: 000020; raw transcriptomic data: https://data.nemoarchive.org/other/grant/AIBS_patchseq/transcriptome/scell/SMARTseq/raw/20200611/. Information for accessing processed data is in the key resources table)
• All original code has been deposited at Zenodo at https://doi.org/10.5281/zenodo.22110698 and is publicly available as of the date of publication. The source_data/figure_plots_with_data_code.md file contains condensed code for accessing source data and plotting figure panels. The code/jan_2026 directory contains all code used in analysis.
• Any additional information required to reanalyze the data reported in this paper is available from the lead contact upon request.

## Acknowledgments

J.G., L.F., and J.M.W. were supported by NIH grant no. U24MH130968. H.S. was supported by NSF grant no. IOS-2216612. We thank all members of the Gillis lab for helpful discussions on the manuscript. J.M.W. gratefully acknowledges the support of Owen White and Anup Mahurkar during the final stages of this work. We also thank Shreejoy Tripathy for helpful discussions on Patch-seq data. The content is solely the responsibility of the authors and does not necessarily represent the official views of the 10.13039/100000002National Institutes of Health.

## Author contributions

J.M.W. and J.G. conceived the project. J.M.W. and J.G. designed the experiments. J.M.W. performed analyses. H.S. created the Figure 1 schematics and performed the ortholog/paralog analyses. L.F. performed the spatial analyses. J.M.W. wrote the manuscript with assistance from J.G. All authors approved the final manuscript.

## Declaration of interests

L.F. owns shares in Quince Therapeutics, serves as a scientific advisor for Lighthouse Pharma, and has received consulting fees from PeopleBio Co. and GC Therapeutics Inc.

## Declaration of generative AI and AI-assisted technologies in the writing process

During the preparation of this work, the authors used ChatGPT in order to edit text for grammar and clarity. After using this tool/service, the authors reviewed and edited the content as needed and take full responsibility for the content of the publication.

## STAR★Methods

### Key resources table

**Table:** 

REAGENT or RESOURCE	SOURCE	IDENTIFIER
**Deposited data**		

Allen Mouse sC Smart-seq V4 Anterior Lateral Motor cortex 2018	Tasic B. et al.^22^	GEO: GSE111157
Allen Mouse sC 10xV2 Primary motor cortex 2021	Yao Z. et al.^18^	NeMO: nemo:dat-ch1nqb7
Allen Mouse sN 10xV2 Primary motor cortex 2021	Yao Z. et al.^18^	NeMO: nemo:dat-ch1nqb7
Allen Mouse sC Smart-seq V4 Primary motor cortex 2021	Yao Z. et al.^18^	NeMO: nemo:dat-ch1nqb7
Allen Mouse sN Smart-seq V4 Primary motor cortex 2021	Yao Z. et al.^18^	NeMO: nemo:dat-ch1nqb7
Allen Mouse sC 10xV2 whole brain atlas 2023	Yao Z. et al.^16^	10*x* RNA-seq data - GEO: GSE246717
Allen Mouse sC 10xV3 whole brain atlas 2023	Yao Z. et al.^16^	10*x* RNA-seq data - GEO: GSE246717
Broad Mouse sC 10xV3 whole brain atlas 2023	Langlieb J et al.^17^	Raw sequencing reads - NeMO: nemo:dat-aa0jwmj; processed data - https://www.braincelldata.org/
Allen cross-primate sN 10xV3 and Smart-seq V4 medial temporal gyrus 2023	Jorstad N. et al.^14^	Raw sequencing reads - NeMO: nemo:dat-net1412; processed data -https://data.nemoarchive.org/publication_release/Great_Ape_MTG_Analysis/
Yale cross-primate sN 10xV3 dorsolateral prefrontal cortex 2022	Ma S. et al.^51^	NeMO: nemo:dat-zkgd0it
UT Southwestern cross-primate sN 10xV3 posterior cingulate cortex 2023	Caglayan E. et al.^52^	GEO: GSE192774
Tree-shrew sN 10xV3 hippocampus 2025	Xiong L. et al.^42^	GEO: GSE229035
Rat sN Parse Biosciences v2 auditory cortex 2024	Gungor Aydin A. et al.^43^	GEO: GSE262970
Rat GEXscope sN cortex	Wang Y. et al.^44^	GEO: GSE253672
Mouse whole brain atlas spatial data	Yao Z. et al.^16^	MERFISH data - https://doi.org/10.35077/g.610
Mouse Patch-seq data	Gouwens N. et al.^20^^,^^53^	raw electrophysiology data - DANDI: 000020 raw transcriptomic data - http://data.nemoarchive.org/other/grant/AIBS_patchseq/transcriptome/scell/SMARTseq/raw/20200611/ Processed data: https://github.com/keon-arbabi/patch-seq-microglia/blob/main/data/processed_ephys/20200625_patchseq_metadata_mouse.csv https://github.com/keon-arbabi/patch-seq-microglia/blob/main/data/processed_ephys/ephys_features_gouwens_w_QC.csv

**Software and algorithms**		

MetaNeighbor	Crow M. et al.^33^	https://github.com/gillislab/MetaNeighbor
MetaMarkers	Fischer S. and Gillis J.^29^	https://github.com/gillislab/MetaMarkers
Code used in all analyses	This paper	https://doi.org/10.5281/zenodo.22110698

### Experimental model and study participant details

This study reuses published data; no new animal or human work was performed and ethics information is provided in the original dataset publications, see associated citations in the *Data collection and processing* Methods Details section.

### Method details

#### Data collection and processing

All data used in this work is publicly available and was sourced as provided by the original authors. CPM normalization was used for all datasets. sC: single-cell, sN: single-nucleus.

Mouse datasets:

• Allen sC Smart-seq V4 2018, Anterior Lateral motor cortex^22^
• Allen sC 10xV2 2021, Primary motor cortex^18^
• Allen sN 10xV2 2021, Primary motor cortex^18^
• Allen sC Smart-seq V4 2021, Primary motor cortex^18^
• Allen sN Smart-seq V4 2021, Primary motor cortex^18^
• Allen sC 10xV2 2023, whole brain atlas^16^
• Allen sC 10xV3 2023, whole brain atlas^16^
• Broad sN 10xV3 2023, whole brain atlas^17^

Cross-primate datasets

• Allen sN 10xV3 and Smart-seq V4 2023, Medial temporal gyrus^14^
• Yale sN 10xV3 2022, Dorsolateral prefrontal cortex^51^
• UT Southwestern sN 10xV3 2023, Posterior cingulate cortex^52^

Tree-shrew dataset

• sN 10xV3 2025, Hippocampus^42^

Rat cortex datasets

• sN Parse Biosciences v2 2024, auditory cortex^43^
• sN GEXscope single nucleus RNA-seq, Singleron Biotechnologies, cortex^44^

For the whole mouse brain atlases, the Broad sN 10xV3 2023 dataset was initially subsetted to clusters with CGE or MGE in their annotation and the Allen sC 10x 2023 datasets were initially subsetted to the CTX-MGE and CTX-CGE class annotated clusters. After the identification of replicable clusters across the 8 mouse datasets (see MetaNeighbor methods section), the replicable mouse clusters were integrated using the Seurat default integration approach (SelectIntegrationFeatures -> FindIntegrationAnchors -> IntegrateData) using default parameters. The integrated cell embedding was used to visualize marker gene expression in Figures 2B–2D, and 2E and the integrated expression data was used to construct the cluster taxonomy across datasets in Figure 2F (see cell-type taxonomies methods section).

The human, chimpanzee, gorilla, macaque, and marmoset MTG dataset was subsetted to the 31 consensus interneuron clusters previously identified across all 5 species.^14^^,^^27^ This consensus subset was used to generate the primate interneuron MetaMarkers (see cell-type marker and MetaMarker generation methods section) and was used in the mouse cluster vs. primate subclass MetaNeighbor assessment. The primate MTG subclass annotations were sourced from Table S6 of the associated publication.^14^ The mouse datasets and the cross-primate MTG dataset were subsetted to 11,228 shared 1-to-1 orthologs across all species before any analysis; orthology information was sourced via Ensembl. All gene names across species were replaced with the human gene name equivalent.

The UT Southwestern cross-primate dataset was subsetted to the 14,649 1-to-1 orthologs identified by the previous authors. *De novo* clusters were computed using the author-provided cross-species integrated shared nearest neighbor graph (Figure S5) with Seurat functions; FindNeighbors(dims = 1:25) and FindClusters(graph.name = ‘integrated_snn’) using default parameters unless otherwise noted.

The Yale cross-primate dataset was subsetted to the 28,216 1-to-1 orthologs identified by the previous authors. The data was subsetted to author-annotated ‘Inh’ cells, split by species, and then integrated using Seurat’s default approach (SelectIntegrationFeatures -> FindIntegrationAnchors -> IntegrateData). *De novo* clusters were computed using the cross-species integrated shared nearest neighbor graph (Figure S5) with Seurat functions; FindNeighbors(dims = 1:25) and FindClusters(graph.name = ‘integrated_snn’) using default parameters unless otherwise noted.

The tree shrew dataset was subsetted to 9,402 1-to-1 orthologs with humans. Cells were filtered to have a minimum of 200 genes and a maximum of 6000 genes detected. Non-neuronal, Inhibitory, and Excitatory cross-primate MetaMarkers from the MTG primate consensus taxonomy^14^^,^^27^ were used to initially annotate individual cells (see cell-type annotation predictions with MetaMarkers methods section). Cells were then filtered for the Inhibitory annotated cells and cells from the author-annotated ‘Infancy’ age annotation were excluded (Figure S12). The data was then split by age (Adult and Aging) and integrated using Seurat’s default approach (SelectIntegrationFeatures -> FindIntegrationAnchors -> IntegrateData). *De novo* clusters were computed using the cross-age integrated shared nearest neighbor graph (Figure S12) with Seurat functions; FindNeighbors(dims = 1:25) and FindClusters(graph.name = ‘integrated_snn’) using default parameters unless otherwise noted.

The Aydin 2024 rat cortex dataset was subsetted to 10,838 1-to-1 orthologs shared with humans and the Wang 2024 rat cortex data was subsetted to 9958 1-to-1 orthologs shared with humans. Both datasets were processed similarly to the tree shrew data above.

All transcriptomic UMAPs were generated with the Seurat function RunUMAP(reduction = ‘pca’, dims = 1:20).

#### MetaNeighbor

MetaNeighbor quantifies the strength of transcriptomic replicability for clusters across datasets using a simple supervised framework, see ref.^33^^,^^34^ and Note S1 for more details. Briefly, a cell-cell similarity graph is constructed across cells being assessed using a set of highly variable genes. We use the top 1000 highly variable genes, identified with the MetaNeighbor variableGenes() function. The cell-type annotations are hidden for a dataset (test dataset) and then predicted by their similarity to the remaining labeled datasets (training data), quantified through the AUROC metric. In the all-vs-all MetaNeighbor procedure, this produces a pairwise assessment of replicability for all the annotated cell-types across all the included datasets. This is implemented with the MetaNeighborUS(fast_version = TRUE) function with default parameters unless noted. The one-vs-best MetaNeighbor procedure first identifies the two closest matching cell types for each test dataset and computes an AUROC quantifying the replicability of the closest match relative to the next best match, as a more specific assessment of replicability between similar clusters. The one-vs-best MetaNeighbor procedure is implemented with the MetaNeighborUS(one_vs_best = TRUE, fast_version = TRUE) function.

To easily identify replicable clusters across datasets, we use a threshold on the reciprocal AUROCs between clusters. If cluster A has an AUROC of 0.95 for cluster B and cluster B has an AUROC of 0.93 for cluster A, then cluster A and B would be reciprocal best matches for an AUROC threshold ≤ 0.93 and neither cluster has a higher-AUROC match to any other cluster. For the cross-mouse dataset MetaNeighbor (Figure S4), we use a reciprocal AUROC threshold of ≥ 0.60 to identify replicable meta-clusters, presented in Table S4. There was a noticeable large group of replicable clusters specific to the whole brain atlas datasets (Figure S4). A large portion of these clusters contained additional regional annotations in their cell-type labels from the Allen sC 10xV2 and 10xV3 whole brain atlas datasets, mostly with striatal tissue annotations (Figure S4). We excluded any mouse meta-cluster that included one of these regionally annotated clusters (NDB-SI-MA-STRv, PAL-STR, STR, RHP-COA) due to these meta-clusters being exclusively comprised of clusters from the whole brain atlas datasets, including the Broad whole brain atlas (Figure S4; Table S4). The included meta-clusters in Table S4 comprise our replicable subset of mouse clusters used in all downstream analysis.

For several of the older mouse datasets when comparing the original subclass labels to our homologous subclass labels, we renamed specific clusters as subclasses, due to their later annotation as subclass-level cell-types. Specifically, Lamp5 Lhx6, Sst Chodl, Pvalb Vipr2, and Serpinf1 were previous clusters that we labeled as Lamp5 Lhx6, Sst Chodl, Chandelier, and Vip subclasses respectively.

We labeled the Broad whole brain atlas clusters with the Allen subclass labels (Table S5) through a majority vote of the meta-clusters in Table S4, these constitute the ‘original mouse subclass’ labels for this dataset. For the one-vs-best MetaNeighbor assessment between the replicable mouse clusters and the cross-primate MTG subclasses (Figure 2A), we first averaged the primate subclass AUROC scores for each mouse cluster and then called mouse clusters as high or low homologous clusters using a threshold of average primate AUROC ≥ 0.60 (Figure S6). The clusters with low homology were excluded and the high homology mouse clusters were manually reordered for the plot in Figure 2A; for each primate subclass, the mouse clusters as columns were ordered by their average primate AUROC, decreasing left to right, and the primate subclasses as rows were ordered phylogenetically.

For the Yale and Southwestern cross-primate MetaNeighbor assessments using the mouse and Allen primate data (Figure S5) we subsetted all datasets to either the 10,637 shared 1-to-1 orthologs present across all the datasets including the Southwestern dataset or the 11,053 shared 1-to-1 orthologs shared when including the Yale dataset.

The graph plots in Figures S5E, S5F, S5K, S5L and S12J, and S12K exhibit 1-vs-best MetaNeighbor AUROCs ≥ 0.60 between clusters.

#### Cell-type marker and MetaMarker generation

MetaMarkers is a differential expression (DE) framework that prioritizes replicable DE across datasets to rank genes as cell-type markers.^29^ DE statistics are first computed for each individual dataset with the compute_markers() function; see ref.^29^ for specific details. Genes are determined as cell-type DE markers for individual datasets using robust DE statistics thresholds, specifically a log2-fold change ≥ 4 and an FDR-adjusted *p*-value ≤ 0.05. The DE statistics are then averaged across all datasets and genes are ranked first by recurrence of DE across datasets and then by the averaged AUROC statistic to settle ties, this is performed with the make_meta_markers() function.

The primate MetaMarkers were generated from the 31 consensus interneuron clusters of the cross-primate MTG dataset using the subclass-level of annotation (Table S1). The mouse MetaMarkers were generated from the 8 mouse datasets using the replicable clusters and their primate-aligned homologous subclass labels (Table S1). The cross-mammal MetaMarkers were generated using the MTG primate datasets and the 8 mouse datasets using their replicable clusters and homologous subclass labels (Table S1).

The marker enrichment score is initially computed on a per-cell basis (see ref.^29^ for more details), we then average this score by cluster for the reported scores in Figures 1E, 1F, 2E, and 2J, and throughout Figures 5, 6, S7, and S12. Let *x*_*ij*_ be the CPM-normalized expression of gene *i* in cell *j*, and *M*_*c*_ be the set of marker genes for cell type *c*. For each cell *j*, we compute a marker score as:

$$S_{j}\;\left(c\right)=\frac{1}{\left|M_{c}\right|}\sum_{i\in M_{c}}\;\log\;2\left(x_{ij}+1\right)$$

This score is the average marker gene expression of a given marker set for each individual cell, implemented by the MetaMarker score_cells() function. We then compute the marker enrichment score by first computing *S*_*j*_(*c*) for a series of cell-types *c*_1_, …,*c*_*n*_ and then compute the following for each cell-type:

$$S_{j}^{\prime }\;\left(c\right)=S_{j}\;\left(c\right)\;/\;\frac{1}{n}\;\sum_{i=1}^{n}S_{j}\;\left(c_{i}\right)$$

This is implemented by the MetaMarker compute_marker_enrichment() function. This normalizes marker set expression in each cell by the average expression of all marker sets, which corresponds to enrichment under the null that all marker sets are expressed equally.

#### Cell-type annotation predictions with MetaMarkers

MetaMarkers uses a simple greedy approach for annotating individual cells using a collection of marker gene sets. First, marker enrichment scores are computed for each cell for a given set of cell-type markers. Then, each cell is annotated with the marker set that has the highest enrichment score; this is implemented through the MetaMarkers assign_cells() function. We use the top 50 primate subclass MetaMarkers (Table S1) to annotate the *de novo* clusters of the Yale and UT Southwestern cross-primate datasets (Figures 5B and 5F).

#### Annotating tree shrew and rat *de novo* clusters

We use the top 200 consensus primate MTG^14^^,^^27^ Inhibitory, Excitatory, and Non-neuronal MetaMarkers when initially annotating the tree shrew dataset. CGE and MGE MetaMarkers were generated using the 5 primate MTG dataset and the 8 mouse datasets using the primate-aligned homologous subclass labels (see MetaNeighbor methods section) and the top 100 CGE/MGE MetaMarkers were used to annotate the Inhibitory tree shrew cells as CGE or MGE (Figure S12). The Vip, Pax6, Sncg, Lamp5, and Lamp5 Lhx6 subclasses were labeled as CGE and the Sst, Sst Chodl, Pvalb, and Chandelier subclasses were labeled as MGE. Clusters with mixed CGE-MGE proportions less than 80:20 (or 20:80) or clusters without clear enrichment of either mouse or primate subclass markers (Figure S12) were excluded and the data was *de novo* clustered again for the final set of 14 clusters as in Figure 6A. These tree-shrew clusters were annotated using a combination of mouse and primate marker enrichments and mouse vs. tree shrew and primate vs. tree shrew 1-vs-best MetaNeighbor assessments (Figure S12). Both rat datasets were processed similarly.

#### Cell-type taxonomies

Hierarchical cell-type taxonomies enable comparisons of transcriptomic similarity across groups of cell-type clusters.^19^^,^^20^^,^^21^^,^^22^ Given a set of cluster annotations, the top 50 markers per cluster are first computed using the MetaMarker compute_markers() function. After filtering the expression data to the set of all top 50 markers across all clusters, the median CPM value per gene per cluster is computed as the expression centroid of each cluster. Spearman correlations are then computed across the cluster expression centroids. Hierarchical clustering using the ward.D2 algorithm is then performed on the centroid correlations, specifically using the hclust(as.dist(1-centroid_spearman), method = ‘ward.D2’) function from the R stats package, with parameters as noted. The mouse data was first filtered for only the high_homology clusters (Figure S6; Table S3) before computing the cell-type cluster taxonomies in Figures 2F and S8. For the integrated mouse taxonomy, we used the intersect of the top 50 markers per cluster per dataset with the 2000 highly variable genes used in the Seurat integration, for a final total of 968 genes used to construct the taxonomy.

#### Computing marker overlaps between homologous cell types

Marker genes from all datasets were mapped to human gene symbols, and the list of paralogous gene pairs in humans were downloaded from Ensembl BioMart v114.^54^^,^^55^ For each pair of *de novo* clusters shared between human and non-human primates in Yale and UT Southwestern datasets, we compared the top 100 markers to determine the frequency of orthologous and paralogous markers expressed in related cell types (Table S6). Markers shared between a pair of clusters were defined as orthologous marker genes. Species-specific markers were identified as the genes uniquely expressed in one species after removing orthologous markers. For each species-specific gene, we retrieved its human paralogs and checked whether any were present in the orthologous marker set or in the other species’ specific markers (Figure 6C). This analysis was performed for all cluster pairs shared between human and non-human primates. The same analysis was repeated in non-primates by comparing human clusters with *de novo* tree-shrew clusters and mouse subclasses (Table S6).

Next, we focused on Vip and Sncg clusters across species to better understand the switch in identity of Yale cluster 17 and UT Southwestern cluster 20 between primates and non-primates. For the human Vip and Sncg clusters in each dataset, we quantified the number of orthologous and paralogous marker genes shared with each Vip and Sncg cluster (in non-human primates and tree-shrew) or subclass (in mouse) in other species (Table S6). These counts were averaged by subclass and the resulting Vip:Sncg ortholog and paralog ratios were visualized to assess patterns of marker conservation and paralog substitution across species (Figure 6E). To determine whether the increased paralog substitution observed in Yale cluster 17 and UT Southwestern cluster 20 was specific to Vip interneurons, we repeated the analysis for Sncg and non-Vip interneuron subclasses between human and mouse (Figure S12L; Table S6).

#### Ion channel transcriptomic data and Patch-seq data

The replicable subset of the mouse datasets with matched primate homology (Table S3) was filtered to include genes present from the following GO terms: GO:0034704 calcium channel complex, GO:0005891 voltage-gated calcium channel complex, GO:0034705 potassium channel complex, GO:0008076 voltage-gated potassium channel complex, GO:0034706 sodium channel complex, GO:0001518 voltage-gated sodium channel complex, and GO:0034707 chloride channel complex, for a total of 176 present genes (Table S2). Data was integrated as described above to produce Figure 3A. The UMAP made using the electrophysiological features (Figure S10B) was generated using the top 10 principal components computed with the stats:princomp() function and the umapumap() function.

Single-cell electrophysiological mouse data was sourced from Gouwens 2020,^20^ which had matched cluster annotations to the Tasic 2018 dataset. Data was made available at^53^

• Cell IDs and their original subclass annotations: https://github.com/keon-arbabi/patch-seq-microglia/blob/main/data/processed_ephys/20200625_patchseq_metadata_mouse.csv
• Processed electrophysiology features: https://github.com/keon-arbabi/patch-seq-microglia/blob/main/data/processed_ephys/ephys_features_gouwens_w_QC.csv

We transferred both original and homologous subclass labels to the electrophysiology data using our homologous mapping in Table S3. In Figure 3, panels D (using the top 2000 highly variable genes) and F (using only the top 25 highly variable genes) use transcriptomic data from Tasic 2018 and panels C and E use the electrophysiological features (*n* = 25) from Gouwens 2020. To compare explained variability between original and homologous subclass labels in panels 3C and 3D, the omega-squared ANOVA effect size was computed as follows:

k=numberofsubclasses

i=1,…,k

$$j=1,\ldots ,n_{i};\;sample\;size\;of\;group\;i$$

$$N=\sum_{i=1}^{k}n_{i};\;total\;sample\;size$$

$$\bar{y_{..}}=\frac{1}{N}\sum_{i=1\;}^{k}\sum_{j=1}^{n_{i}}y_{ij};\;grand\;mean$$

$$\bar{y_{i.}}=\frac{1}{n_{i}}\sum_{j=1}^{n_{i}}y_{ij};group\;mean$$

$${SS}_{total}=\sum_{i=1}^{k}\sum_{j=1}^{n_{i}}{\left(y_{ij}-\bar{y_{..}}\right)}^{2}$$

$${SS}_{between}=\sum_{i=1}^{k}n_{i}{\left(\bar{y_{i.}}-\bar{y_{..}}\right)}^{2}$$

$${SS}_{within}={SS}_{total}-{SS}_{between}$$

$${MS}_{within}=\frac{{SS}_{within}}{N-k}$$

$$\omega^{2}=\frac{{SS}_{between}-\left(k-1\right){MS}_{within}}{{SS}_{total}+{MS}_{within}},\text{omega}-\text{squared}$$

Electrophysiological features and gene features were all z-scored before computing omega-squared values, using the R scale() function.

To identify electrophysiological features that distinguish newly annotated Pax6 cells from their previous subclass annotations, we utilized an empirical modeling approach. For a given feature, an upper and lower probability value was computed for each Pax6 cell given that cell’s rank within the distribution of cells with stable annotations across species (all other CGE cells in Figures 3G and 3H, Lamp5 cells that remain annotated as Lamp5 between mice and primates for Figures S10C and S10D, and Vip cells that remain annotated as Vip for Figures S10E and S10F). A two-sided probability value is computed as 2∗minimum(p_lower, p_upper). This provides a distribution of *p*-values for each electrophysiological feature. We compute and report an aggregate *p*-value per feature (Figures 3G, S10C, and S10E) using Fisher’s Method to combine *p*-values, where the aggregate *p*-value is given through the Chi-squared distribution:

$X_{2k}^{2}=-2\sum_{i=1}^{k}\ln\;p_{i}$, where k = the number of Pax6 cells or number of *p*-values per feature.

Specifically, the aggregate *p*-value was computed using the R function pchisq(chi-statistic, 2∗k, lower.tail = FALSE), where chi-statistic = $X_{2k}^{2}$.

#### Spatial data

Mouse spatial data was sourced from.^16^ Target clusters (columns of Figures 4A–4C and S11) were filtered using the CTX-CGE GABA and CTX-MGE GABA class labels. For the spatial data, we kept the Lamp5 Lhx6 clusters labeled as MGE to align with the spatial annotations, though we note that in many of the transcriptional datasets, Lamp5 Lhx6 clusters are annotated as a CGE cell-type. We compute the cell-type neighbor proportion using the top 100 nearest neighbors per cell within the isocortex or hippocampal formation parcellations, taking the average across cells for a cluster. Cell groupings with fewer than 100 cells after the regional filtering were excluded from neighborhood analyses. Layer proportions of our target clusters (Figure 4C) were computed using the Common Coordinate Framework (CCFv3) layer annotations outlined in the annotated layers schematic adjacent to Figure 4C.^56^ Hierarchical clustering was performed using the ward.D2 algorithm in R and heatmaps were generated using the ComplexHeatmap R package.

### Quantification and statistical analysis

#### Software

All analysis was performed in R v4.4.3. Single-cell data was analyzed using either Seurat^57^ v5.0.0 objects or SingleCellExperiment^58^ v1.26.0 objects. Data visualization was performed with ggplot2 and ComplexHeatmap. All ggplot boxplot elements are the same across figure panels; the center line depicts the median, the lower and upper box limits depict the 1^st^ and 3^rd^ quartiles, and the lower and upper whiskers extend to the most extreme data points within the Q1 – 1.5∗IQR or Q3 + 1.5∗IQR respectively.

#### Statistical analysis

All reported data points with error bars are mean values ± standard deviations. Details for AUROC metrics, the omega-squared ANOVA effect size computation, and Fisher’s Method for combining *p*-values are provided in the Methods details section.

## Supplemental information

Document S1. Figures S1–S13 and Data S1/Methods S1

Table S1. Cross-species MetaMarker differential expression statistics MetaMarker statistics for the primate MetaMarkers, mouse MetaMarkers, and the cross-mammal (mouse and primate datasets) MetaMarkers. Genes are initially ranked by recurrence of differential expression across datasets, then ranked by the AUROC statistic to break ties

Table S2. Ion channel GO terms List of present ion channel genes across our 8 mouse datasets from the GO terms GO:0034704, GO:0034705, GO:0034706, GO:0034707, GO:0001518, GO:0005891, GO:0008076; 176 unique genes

Table S3. Original and homologous cluster annotations Original author provided cluster annotations for all mouse datasets with the homologous primate subclass annotations and whether the cluster is classified as having high or low homology (see MetaNeighbor methods section)

Table S4. MetaNeighbor meta-cluster annotations MetaNeighbor meta-cluster assignments for all mouse clusters. The excluded meta-clusters (see MetaNeighbor methods section) and the ‘outliers’ meta-cluster were omitted from analysis

Table S5. Broad and Allen mouse cluster annotation alignment Mapping between the Broad cluster annotations and the original Allen subclass annotations

Table S6. Cross-species ortholog and paralog overlaps Summaries of ortholog and paralog overlaps between species cluster comparisons. The ‘summary_of_shared_markers’ sheet is the ortholog and paralog percentage overlap of the top 100 markers per cluster. The ‘list_of_shared_markers’ sheet is the ortholog and paralog designation for genes. The ‘Vip_Sncg_mark_summary’ contains the overlaps from which the ratios in Figure 6E are derived. The ‘Vip_Sncg_marker_list’ contains the ortholog and paralog designations for the Vip and Sncg clusters. The ‘human_mouse_Sncg_marker_overlap’ contains the ortholog and paralog overlaps between human and mouse cluster comparisons as in Figure S11L

## References

[1] Ochi S., Manabe S., Kikkawa T., Osumi N. (2022). Thirty Years’ History since the Discovery of Pax6: From Central Nervous System Development to Neurodevelopmental Disorders. Int. J. Mol. Sci..

[2] Hill R.E., Favor J., Hogan B.L., Ton C.C., Saunders G.F., Hanson I.M., Prosser J., Jordan T., Hastie N.D., van Heyningen V. (1991). Mouse Small eye results from mutations in a paired-like homeobox-containing gene. Nature.

[3] Kozmik Z. (2005). Pax genes in eye development and evolution. Curr. Opin. Genet. Dev..

[4] Onuma Y., Takahashi S., Asashima M., Kurata S., Gehring W.J. (2002). Conservation of Pax 6 function and upstream activation by Notch signaling in eye development of frogs and flies. Proc. Natl. Acad. Sci..

[5] Glaser T., Jepeal L., Edwards J.G., Young S.R., Favor J., Maas R.L. (1994). PAX6 gene dosage effect in a family with congenital cataracts, aniridia, anophthalmia and central nervous system defects. Nat. Genet..

[6] Simpson T.I., Price D.J. (2002). A pleiotropic player in development. Bioessays.

[7] Bishop K.M., Goudreau G., O’Leary D.D.M. (2000). Regulation of Area Identity in the Mammalian Neocortex by Emx2 and Pax6. Science.

[8] Osumi N., Shinohara H., Numayama-Tsuruta K., Maekawa M. (2008). Concise Review: Pax6 Transcription Factor Contributes to both Embryonic and Adult Neurogenesis as a Multifunctional Regulator. Stem Cell..

[9] Englund C., Fink A., Lau C., Pham D., Daza R.A.M., Bulfone A., Kowalczyk T., Hevner R.F. (2005). Pax6, Tbr2, and Tbr1 Are Expressed Sequentially by Radial Glia, Intermediate Progenitor Cells, and Postmitotic Neurons in Developing Neocortex. J. Neurosci..

[10] Jorstad N.L., Close J., Johansen N., Yanny A.M., Barkan E.R., Travaglini K.J., Bertagnolli D., Campos J., Casper T., Crichton K. (2023). Transcriptomic cytoarchitecture reveals principles of human neocortex organization. Science.

[11] Siletti K., Hodge R., Mossi Albiach A., Lee K.W., Ding S.L., Hu L., Lönnerberg P., Bakken T., Casper T., Clark M. (2023). Transcriptomic diversity of cell types across the adult human brain. Science.

[12] Gabitto M.I., Travaglini K.J., Rachleff V.M., Kaplan E.S., Long B., Ariza J., Ding Y., Mahoney J.T., Dee N., Goldy J. (2024). Integrated multimodal cell atlas of Alzheimer’s disease. Nat. Neurosci..

[13] Johansen N., Somasundaram S., Travaglini K.J., Yanny A.M., Shumyatcher M., Casper T., Cobbs C., Dee N., Ellenbogen R., Ferreira M. (2023). Interindividual variation in human cortical cell type abundance and expression. Science.

[14] Jorstad N.L., Song J.H.T., Exposito-Alonso D., Suresh H., Castro-Pacheco N., Krienen F.M., Yanny A.M., Close J., Gelfand E., Long B. (2023). Comparative transcriptomics reveals human-specific cortical features. Science.

[15] Wei J.-R., Hao Z.Z., Xu C., Huang M., Tang L., Xu N., Liu R., Shen Y., Teichmann S.A., Miao Z., Liu S. (2022). Identification of visual cortex cell types and species differences using single-cell RNA sequencing. Nat. Commun..

[16] Yao Z., van Velthoven C.T.J., Kunst M., Zhang M., McMillen D., Lee C., Jung W., Goldy J., Abdelhak A., Aitken M. (2023). A high-resolution transcriptomic and spatial atlas of cell types in the whole mouse brain. Nature.

[17] Langlieb J., Sachdev N.S., Balderrama K.S., Nadaf N.M., Raj M., Murray E., Webber J.T., Vanderburg C., Gazestani V., Tward D. (2023). The molecular cytoarchitecture of the adult mouse brain. Nature.

[18] Yao Z., Liu H., Xie F., Fischer S., Adkins R.S., Aldridge A.I., Ament S.A., Bartlett A., Behrens M.M., Van den Berge K. (2021). A transcriptomic and epigenomic cell atlas of the mouse primary motor cortex. Nature.

[19] Scala F., Kobak D., Bernabucci M., Bernaerts Y., Cadwell C.R., Castro J.R., Hartmanis L., Jiang X., Laturnus S., Miranda E. (2021). Phenotypic variation of transcriptomic cell types in mouse motor cortex. Nature.

[20] Gouwens N.W., Sorensen S.A., Baftizadeh F., Budzillo A., Lee B.R., Jarsky T., Alfiler L., Baker K., Barkan E., Berry K. (2020). Integrated Morphoelectric and Transcriptomic Classification of Cortical GABAergic Cells. Cell.

[21] Yao Z., van Velthoven C.T.J., Nguyen T.N., Goldy J., Sedeno-Cortes A.E., Baftizadeh F., Bertagnolli D., Casper T., Chiang M., Crichton K. (2021). A taxonomy of transcriptomic cell types across the isocortex and hippocampal formation. Cell.

[22] Tasic B., Yao Z., Graybuck L.T., Smith K.A., Nguyen T.N., Bertagnolli D., Goldy J., Garren E., Economo M.N., Viswanathan S. (2018). Shared and distinct transcriptomic cell types across neocortical areas. Nature.

[23] Tosches M.A., Yamawaki T.M., Naumann R.K., Jacobi A.A., Tushev G., Laurent G. (2018). Evolution of pallium, hippocampus, and cortical cell types revealed by single-cell transcriptomics in reptiles. Science.

[24] Shafer M.E.R. (2019). Cross-Species Analysis of Single-Cell Transcriptomic Data. Front. Cell Dev. Biol..

[25] Shafer M.E.R., Sawh A.N., Schier A.F. (2022). Gene family evolution underlies cell type diversification in the hypothalamus of teleosts. Nat. Ecol. Evol..

[26] Bakken T.E., Jorstad N.L., Hu Q., Lake B.B., Tian W., Kalmbach B.E., Crow M., Hodge R.D., Krienen F.M., Sorensen S.A. (2021). Comparative cellular analysis of motor cortex in human, marmoset and mouse. Nature.

[27] Suresh H., Crow M., Jorstad N., Hodge R., Lein E., Dobin A., Bakken T., Gillis J. (2023). Comparative single-cell transcriptomic analysis of primate brains highlights human-specific regulatory evolution. Nat. Ecol. Evol..

[28] Bakken T.E., Hodge R.D., Miller J.A., Yao Z., Nguyen T.N., Aevermann B., Barkan E., Bertagnolli D., Casper T., Dee N. (2018). Single-nucleus and single-cell transcriptomes compared in matched cortical cell types. PLoS One.

[29] Fischer S., Gillis J. (2021). How many markers are needed to robustly determine a cell’s type?. iScience.

[30] French L., Suresh H., Gillis J. (2026). Cluster replicability in single-cell and single-nucleus atlases of the mouse brain. Nat. Commun..

[31] Hodge R.D., Bakken T.E., Miller J.A., Smith K.A., Barkan E.R., Graybuck L.T., Close J.L., Long B., Johansen N., Penn O. (2019). Conserved cell types with divergent features in human versus mouse cortex. Nature.

[32] Chartrand T., Dalley R., Close J., Goriounova N.A., Lee B.R., Mann R., Miller J.A., Molnar G., Mukora A., Alfiler L. (2023). Morphoelectric and transcriptomic divergence of the layer 1 interneuron repertoire in human versus mouse neocortex. Science.

[33] Crow M., Paul A., Ballouz S., Huang Z.J., Gillis J. (2018). Characterizing the replicability of cell types defined by single cell RNA-sequencing data using MetaNeighbor. Nat. Commun..

[34] Fischer S., Crow M., Harris B.D., Gillis J. (2021). Scaling up reproducible research for single-cell transcriptomics using MetaNeighbor. Nat. Protoc..

[35] Taniguchi H., Lu J., Huang Z.J. (2013). The Spatial and Temporal Origin of Chandelier Cells in Mouse Neocortex. Science.

[36] Gao Y., van Velthoven C.T.J., Lee C., Thomas E.D., Mathieu R., Ayala A.P., Barta S., Bertagnolli D., Campos J., Cardenas T. (2025). Continuous cell-type diversification in mouse visual cortex development. Nature.

[37] Miyoshi G., Hjerling-Leffler J., Karayannis T., Sousa V.H., Butt S.J.B., Battiste J., Johnson J.E., Machold R.P., Fishell G. (2010). Genetic Fate Mapping Reveals That the Caudal Ganglionic Eminence Produces a Large and Diverse Population of Superficial Cortical Interneurons. J. Neurosci..

[38] Han L., Liu Z., Jing Z., Liu Y., Peng Y., Chang H., Lei J., Wang K., Xu Y., Liu W. (2025). Single-cell spatial transcriptomic atlas of the whole mouse brain. Neuron.

[39] Wei S., Luo M., Wang P., Chen R., Jin X., Xu C., Li C., Lin X., Xu Z., Liu H. (2025). Charting the spatial transcriptome of the human cerebral cortex at single-cell resolution. Nat. Commun..

[40] Langseth C.M., Gyllborg D., Miller J.A., Close J.L., Long B., Lein E.S., Hilscher M.M., Nilsson M. (2021). Comprehensive in situ mapping of human cortical transcriptomic cell types. Commun. Biol..

[41] Zhang M., Eichhorn S.W., Zingg B., Yao Z., Cotter K., Zeng H., Dong H., Zhuang X. (2021). Spatially resolved cell atlas of the mouse primary motor cortex by MERFISH. Nature.

[42] Xiong L.-L., Niu R.Z., Chen L., Huangfu L.R., Li J., Xue L.L., Sun Y.F., Wang L.M., Li Y.P., Liu J., Wang T.H. (2025). Cross-Species Insights from Single-Nucleus Sequencing Highlight Aging-Related Hippocampal Features in Tree Shrew. Mol. Biol. Evol..

[43] Gungor Aydin A., Lemenze A., Bieszczad K.M. (2024). Functional diversities within neurons and astrocytes in the adult rat auditory cortex revealed by single-nucleus RNA sequencing. Sci. Rep..

[44] Wang Y., Ma Y., Zhong Q., Song B., Liu Q. (2024). Transcriptomic analysis of rat brain response to alternating current electrical stimulation: unveiling insights via single-nucleus RNA sequencing. MedComm.

[45] Harris B.D., Crow M., Fischer S., Gillis J. (2021). Single-cell co-expression analysis reveals that transcriptional modules are shared across cell types in the brain. Cell Syst..

[46] Yuste R., Hawrylycz M., Aalling N., Aguilar-Valles A., Arendt D., Armañanzas R., Ascoli G.A., Bielza C., Bokharaie V., Bergmann T.B. (2020). A community-based transcriptomics classification and nomenclature of neocortical cell types. Nat. Neurosci..

[47] Zeng H. (2022). What is a cell type and how to define it?. Cell.

[48] Kiselev V.Y., Andrews T.S., Hemberg M. (2019). Challenges in unsupervised clustering of single-cell RNA-seq data. Nat. Rev. Genet..

[49] Arendt D., Bertucci P.Y., Achim K., Musser J.M. (2019). Evolution of neuronal types and families. Curr. Opin. Neurobiol..

[50] Tosches M.A. (2021). From Cell Types to an Integrated Understanding of Brain Evolution: The Case of the Cerebral Cortex. Annu. Rev. Cell Dev. Biol..

[51] Ma S., Skarica M., Li Q., Xu C., Risgaard R.D., Tebbenkamp A.T.N., Mato-Blanco X., Kovner R., Krsnik Ž., de Martin X. (2022). Molecular and cellular evolution of the primate dorsolateral prefrontal cortex. Science.

[52] Caglayan E., Ayhan F., Liu Y., Vollmer R.M., Oh E., Sherwood C.C., Preuss T.M., Yi S.V., Konopka G. (2023). Molecular features driving cellular complexity of human brain evolution. Nature.

[53] Arbabi K., Jiang Y., Howard D., Nigam A., Inoue W., Gonzalez-Burgos G., Felsky D., Tripathy S.J. (2023). Investigating microglia-neuron crosstalk by characterizing microglial contamination in human and mouse patch-seq datasets. iScience.

[54] Herrero J., Muffato M., Beal K., Fitzgerald S., Gordon L., Pignatelli M., Vilella A.J., Searle S.M.J., Amode R., Brent S. (2016). Ensembl comparative genomics resources. Ensembl comparative genomics resources.

[55] Dyer S.C., Austine-Orimoloye O., Azov A.G., Barba M., Barnes I., Barrera-Enriquez V.P., Becker A., Bennett R., Beracochea M., Berry A. (2025). Ensembl 2025. Nucleic Acids Res..

[56] Wang Q., Ding S.L., Li Y., Royall J., Feng D., Lesnar P., Graddis N., Naeemi M., Facer B., Ho A. (2020). The Allen Mouse Brain Common Coordinate Framework: A 3D Reference Atlas. Cell.

[57] Hao Y., Stuart T., Kowalski M.H., Choudhary S., Hoffman P., Hartman A., Srivastava A., Molla G., Madad S., Fernandez-Granda C., Satija R. (2024). Dictionary learning for integrative, multimodal and scalable single-cell analysis. Nat. Biotechnol..

[58] Amezquita R.A., Lun A.T.L., Becht E., Carey V.J., Carpp L.N., Geistlinger L., Marini F., Rue-Albrecht K., Risso D., Soneson C. (2020). Orchestrating single-cell analysis with Bioconductor. Nat. Methods.

