# Supplementary material for: The misplaced mouse Pax6 interneuron subclass: A cross-species transcriptomic reassignment
Source: iScience. 2026 Sep 26;29(10):117632. doi: 10.1016/j.isci.2026.117632 (PMC13639153; doi:10.1016/j.isci.2026.117632)
Supplement: Supplementary file 1 [file mmc1.pdf]

iScience, Volume *29*

Supplemental information

The misplaced mouse Pax6 interneuron subclass: A cross-species transcriptomic reassignment

Jonathan M. Werner, Hamsini Suresh, Leon French, and Jesse Gillis

Data S1/Methods S1: one-vs-best MetaNeighbor scores

For detailed information on the MetaNeighbor protocols, see reference 33 (Fischer 2021). MetaNeighbor quantifies the transcriptional replicability of clusters across independent datasets using a neighbor-voting framework. Cells in a reference dataset rank cells in a test dataset based on transcriptional similarity, and this ranking is converted into a cluster-label prediction task using the AUROC statistic.

For example, consider a case where cluster A is the reference cluster, and we are testing the replicability of clusters X, Y, and Z in a test dataset. If cluster A and cluster Y represent the same cell type across the two datasets, their transcriptional profiles are expected to be highly similar. An AUROC score of 0.90 between cluster A and cluster Y indicates that, using cluster A as the scoring reference, a randomly chosen cell from cluster Y is ranked ahead of a randomly chosen cell not in cluster Y 90% of the time. In other words, the transcriptional profile of cluster A is a strong predictor of the transcriptional profile of cluster Y, reflecting strong transcriptional similarity. In the standard MetaNeighbor protocol, all clusters between a reference and test dataset are compared pairwise, yielding an AUROC value for every cluster comparison.

The one-vs-best MetaNeighbor protocol first identifies the top two matching test clusters for a given reference cluster, and then evaluates how strongly the reference cluster distinguishes between those two matches using the AUROC score. This analysis yields two broad outcomes, both of which are represented in our results.

In the first scenario, the top two matches are transcriptionally distinct, and there is a clear best match for the reference cluster. In this case, the reference cluster preferentially ranks the top match above the second-best match, producing an AUROC > 0.50 for the top match and an AUROC < 0.50 for the second match, indicating that cells from the latter cluster fall near the bottom of the ranked list. This pattern is observed for the mouse–primate cluster matches in Figure 2A, where each mouse cluster has a clear top primate match (AUROCs > 0.50; red signal in the heatmap) that is distinct from its next-best primate match (AUROCs < 0.50; blue signal in the heatmap).

In the second scenario, the top two test matches are both reasonable matches for the reference cluster, either because all three clusters are transcriptionally similar or because the reference cluster shares features with both. In this case, the reference cluster fails to preferentially predict one test cluster over the other, producing AUROC values near 0.50. We describe these as clusters with low single-subclass homology across species, referred to as the “low homology” clusters in Supplementary Table 3 and Supplementary Figure 6.

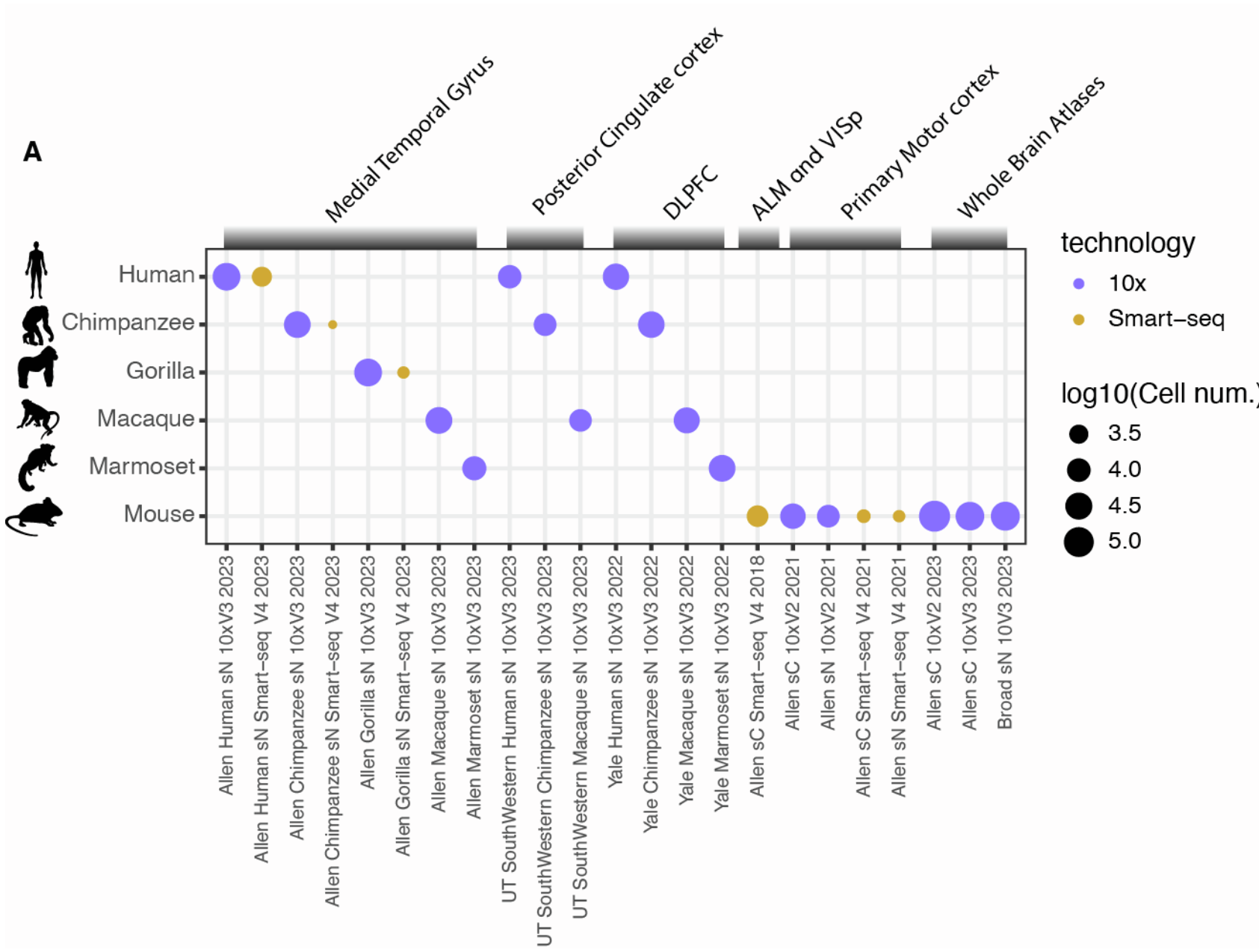

Supplemental Figure 1: Mouse and cross-primate interneuron datasets

A: Bubble plot depicting the cell number of each dataset, including the species, brain region, and single-cell technology of each dataset. sN: single-nucleus, sC: single-cell.

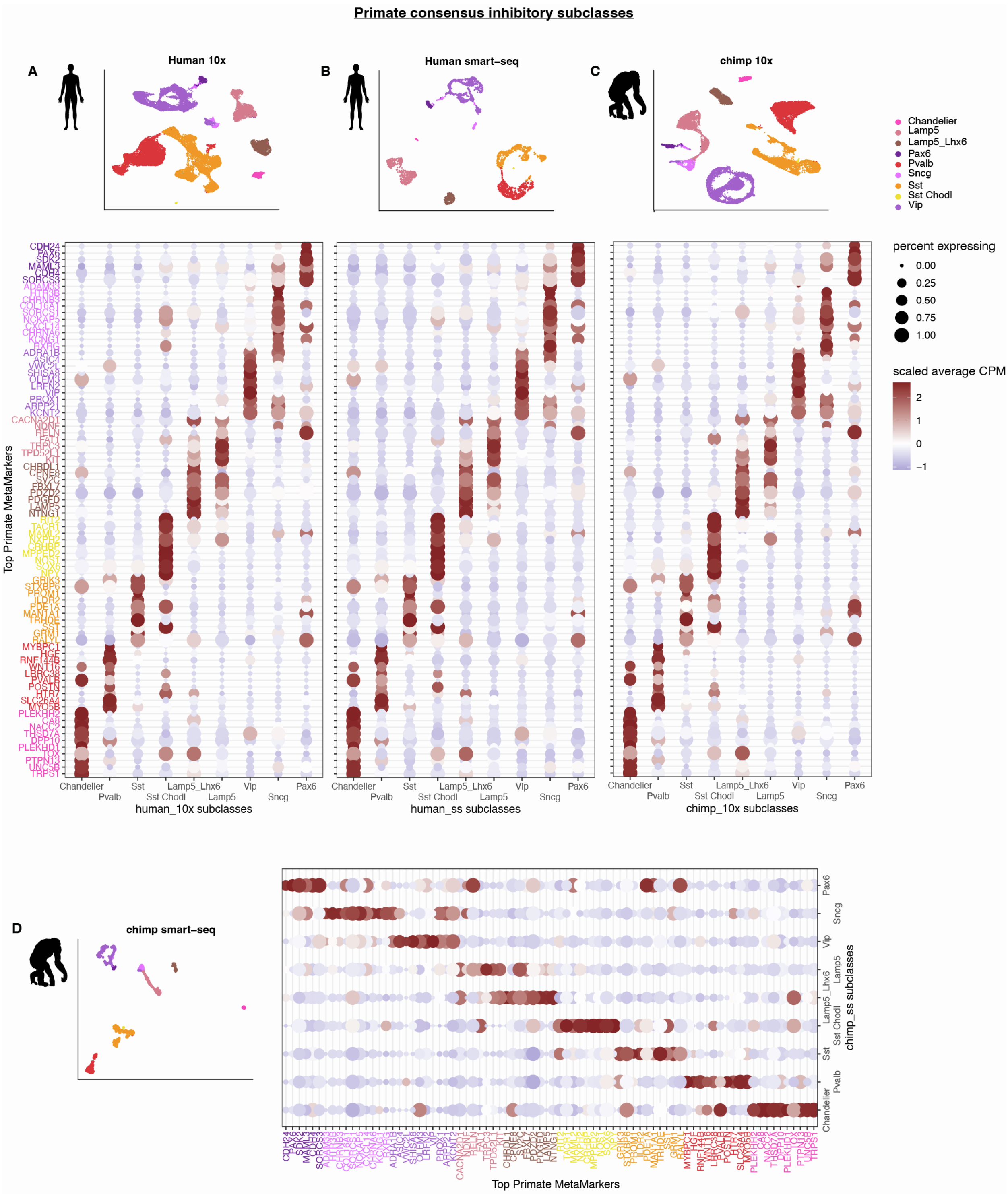

Primate consensus inhibitory subclasses
A
Human 10x
B
Human smart-seq
C
chimp 10x
D
chimp smart-seq
Chandelier
Lamp5
Lamp5_Lhx6
Pax6
Pvalb
Sncg
Sst
Sst Chodl
Vip
percent expressing
0.00
0.25
0.50
0.75
1.00
scaled average CPM
2
1
0
−1
Top Primate MetaMarkers
human_10x subclasses
human_ss subclasses
chimp_10x subclasses
chimp_ss subclasses

Supplemental Figure 2: Consensus cross-primate interneuron subclass marker gene expression for human and chimpanzee datasets

A: UMAP depicting the cross-primate consensus subclass annotations for the human MTG 10x dataset. Bubble plot depicting the z-scored CPM values (color) and the percentage of cells expressing (CPM > 0, dot size) the top 10 primate subclass MetaMarkers (Supp. Table 1) across the subclass annotations for the human MTG 10X dataset. Duplicate MetaMarkers across the top 10 are colored for visualization purposes.
B: Same as in **A**, but for the human Smart-seq MTG dataset.
C: Same as in **A**, but for the chimpanzee 10x MTG dataset.
D: Same as in **A**, but for the chimpanzee Smart-seq MTG dataset.

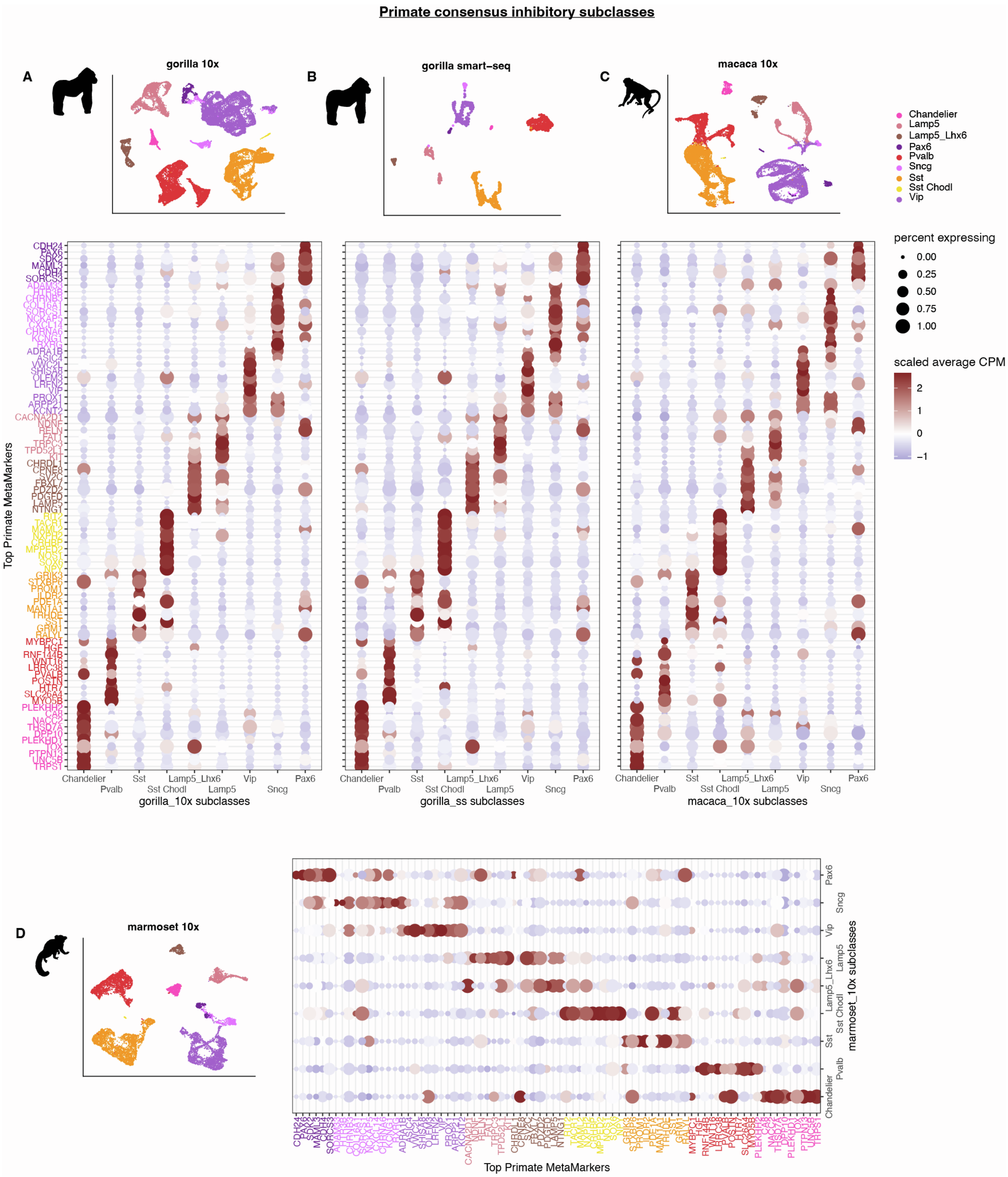

Primate consensus inhibitory subclasses
A
gorilla 10x
B
gorilla smart−seq
C
macaca 10x
Chandelier
Lamp5
Lamp5_Lhx6
Pax6
Pvalb
Sncg
Sst
Sst Chodl
Vip
percent expressing
0.00
0.25
0.50
0.75
1.00
scaled average CPM
2
1
0
−1
Top Primate MetaMarkers
Chandelier
Pvalb
Sst
Sst Chodl
Lamp5_Lhx6
Lamp5
Vip
Sncg
Pax6
gorilla_10x subclasses
gorilla_ss subclasses
macaca_10x subclasses
D
marmoset 10x
marmoset_10x subclasses
Top Primate MetaMarkers

Supplemental Figure 3: Consensus cross-primate interneuron subclass marker gene expression for gorilla, macaque, and marmoset datasets

A: UMAP depicting the cross-primate consensus subclass annotations for the gorilla MTG 10x dataset. Bubble depicting the z-scored CPM values (color) and the percentage of cells expressing (CPM > 0, dot size) the top 10 primate subclass MetaMarkers (Supp. Table 1) across the subclass annotations for the gorilla MTG 10X dataset. Duplicate MetaMarkers across the top 10 are colored for visualization purposes.

B: Same as in **A**, but for the gorilla Smart-seq MTG dataset.

C: Same as in **A**, but for the macaque 10x MTG dataset.

D: Same as in **A**, but for the marmoset 10x MTG dataset.

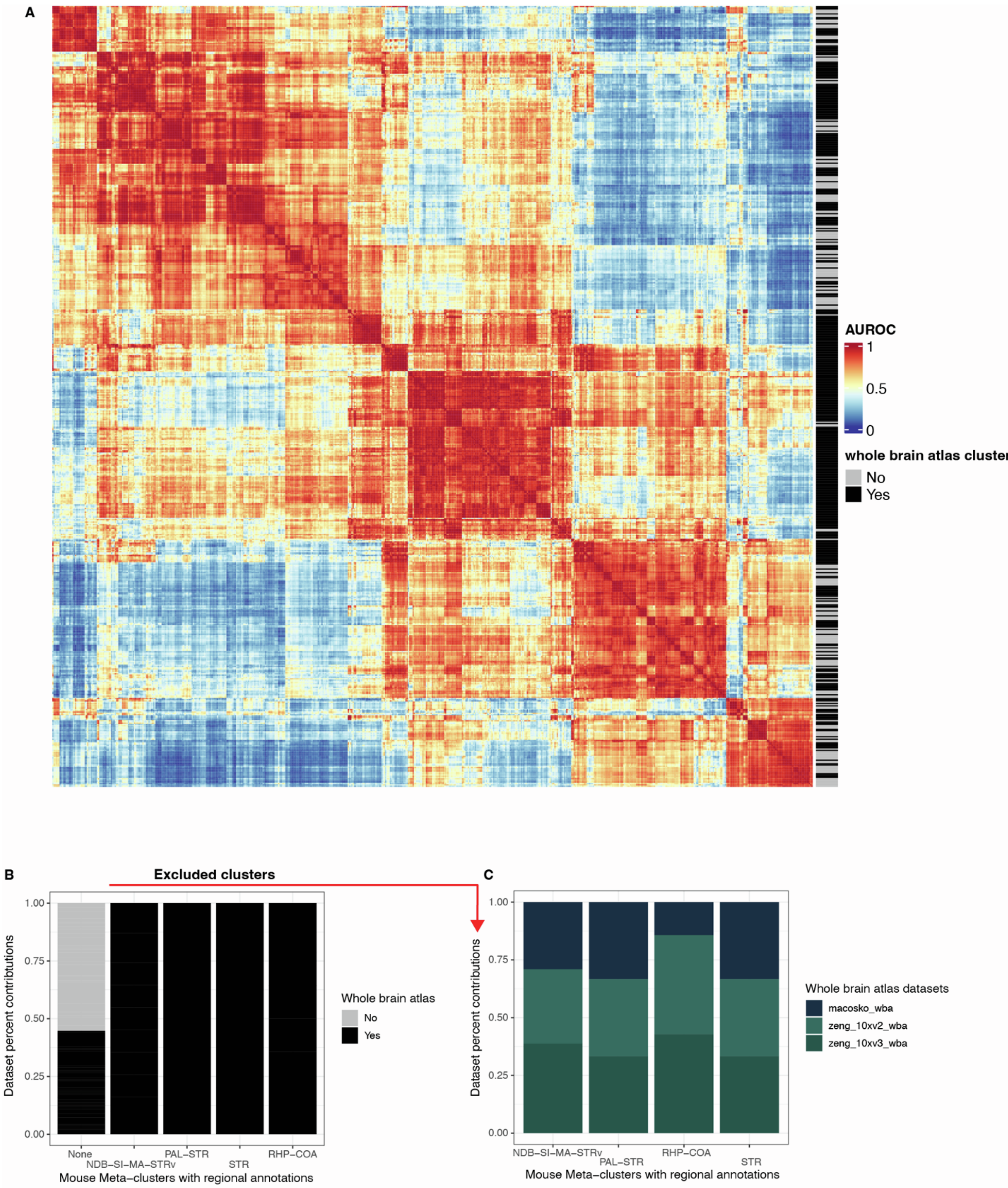

A
AUROC
1
0.5
0
whole brain atlas cluster
No
Yes
B
Excluded clusters
Dataset percent contributions
1.00
0.75
0.50
0.25
0.00
None
NDB−SI−MA−STRv
PAL−STR
STR
RHP−COA
Mouse Meta−clusters with regional annotations
Whole brain atlas
No
Yes
C
Dataset percent contributions
1.00
0.75
0.50
0.25
0.00
NDB−SI−MA−STRv
PAL−STR
RHP−COA
STR
Mouse Meta−clusters with regional annotations
Whole brain atlas datasets
macosko_wba
zeng_10xv2_wba
zeng_10xv3_wba

Supplemental Figure 4: Replicability of mouse clusters

A: Heatmap depicting the all-vs-all MetaNeighbor AUROC scores for all clusters across the 8 mouse datasets. The row annotation denotes whether that cluster belongs to one of the whole brain mouse datasets. Note the large collection of replicable clusters nearly exclusively from the whole brain atlas datasets.
B: Stacked barplots depicting the dataset contributions (whole brain atlas or not) of mouse meta-clusters (see *MetaNeighbor* methods section) that include clusters with either NDB-SI-MA-STRv, PAL-STR, STR, RHP-COA or none of these regional annotations. The meta-clusters that include those regional annotations are exclusively composed of clusters from the whole-brain datasets.
C: Stacked barplots depicting the dataset contributions (dataset labels) of those meta-clusters that include the regional annotations. All 3 whole brain datasets contribute to these meta-clusters, and these clusters are excluded from the mouse datasets for all downstream analysis.

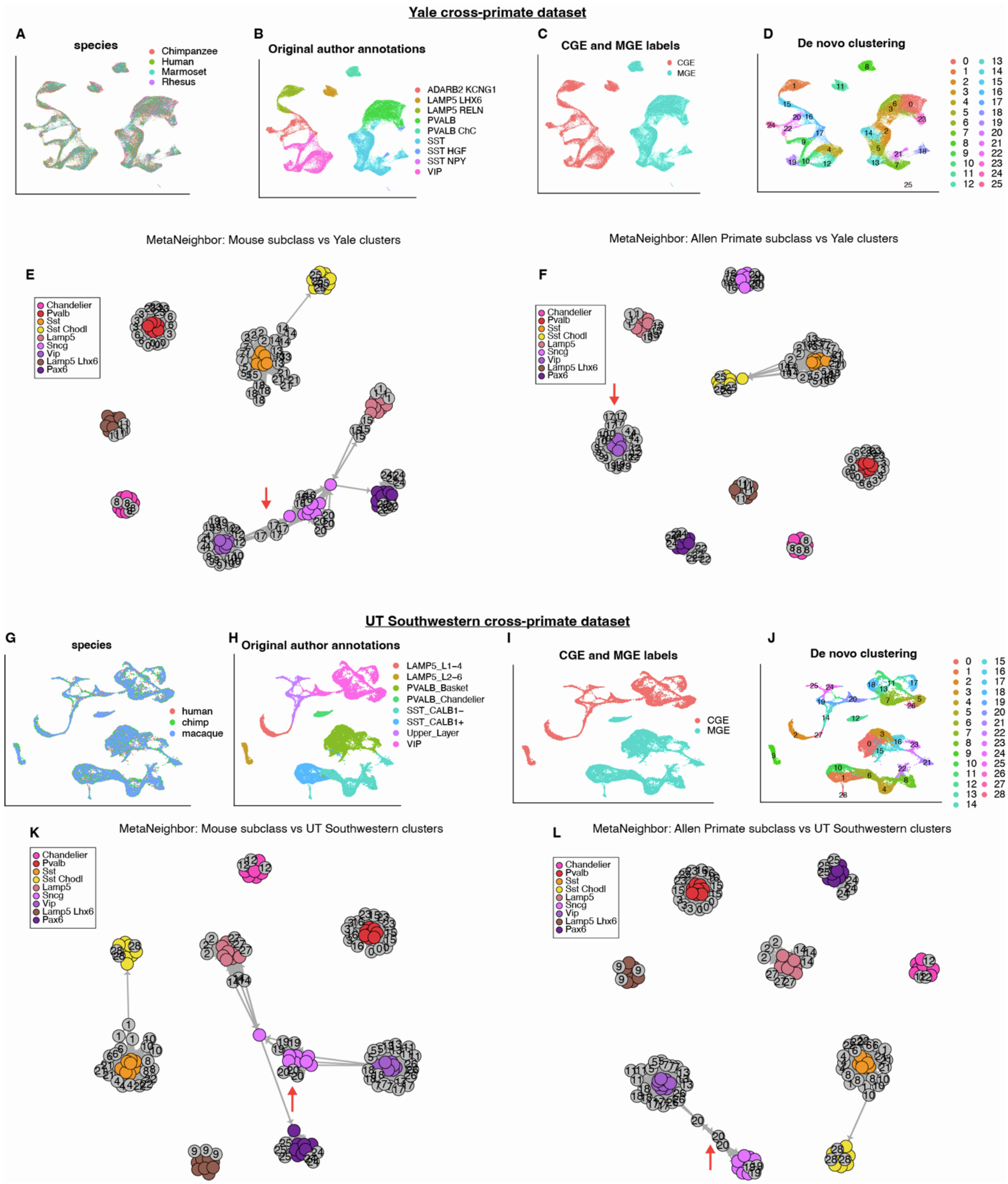

Yale cross-primate dataset
A
species
Chimpanzee
Human
Marmoset
Rhesus
B
Original author annotations
ADARB2 KCNG1
LAMP5 LHX6
LAMP5 RELN
PVALB
PVALB ChC
SST
SST HGF
SST NPY
VIP
C
CGE and MGE labels
CGE
MGE
D
De novo clustering
MetaNeighbor: Mouse subclass vs Yale clusters
MetaNeighbor: Allen Primate subclass vs Yale clusters
E
F
Chandelier
Pvalb
Sst
Sst Chodl
Lamp5
Sncg
Vip
Lamp5 Lhx6
Pax6
UT Southwestern cross-primate dataset
G
species
human
chimp
macaque
H
Original author annotations
LAMP5_L1-4
LAMP5_L2-6
PVALB_Basket
PVALB_Chandelier
SST_CALB1−
SST_CALB1+
Upper_Layer
VIP
I
CGE and MGE labels
CGE
MGE
J
De novo clustering
K
MetaNeighbor: Mouse subclass vs UT Southwestern clusters
L
MetaNeighbor: Allen Primate subclass vs UT Southwestern clusters

Supplemental Figure 5: Yale and UT Southwestern cross-primate dataset de novo clustering and subclass MetaNeighbor assessments

A: UMAP depicting the integrated cross-species data for the Yale dataset, colored by species.
B: Same UMAP as in **A**, colored by the authors original cell-type annotations.
C: Same UMAP as in **A**, colored by the CGE and MGE classification of the author cell-type annotations.
D: Same UMAP as in **A**, depicting the de novo clusters of the Yale dataset.
E: Graph plot depicting the one-vs-best MetaNeighbor scores between the mouse homologous subclasses and the Yale de novo clusters. Edges denote AUROCs >= 0.60, edge length corresponds inversely to AUROCs (high AUROCs -> small edges). The red arrow highlights cluster 17, which has intermediate primate Vip/mouse Sncg characteristics (from Figure 5).
F: Same as in panel **E**, but depicting the MetaNeighbor scores between the Allen MTG primate subclasses and the Yale de novo clusters.
G: UMAP depicting the integrated cross-species data for the UT Southwestern dataset, colored by species.
H: Same UMAP as in **G**, colored by the authors original cell-type annotations.
I: Same UMAP as in **G**, colored by the CGE and MGE classification of the author cell-type annotations.
J: Same UMAP as in **G**, depicting the de novo clusters of the UT Southwestern dataset.
K: Graph plot depicting the one-vs-best MetaNeighbor scores between the mouse homologous subclasses and the UT Southwestern de novo clusters. Edges denote AUROCs >= 0.60. The red arrow highlights cluster 20, which has intermediate primate Vip/mouse Sncg characteristics (from Figure 5).
L: Same as in panel **K**, but depicting the MetaNeighbor scores between the Allen MTG primate subclasses and the UT Southwestern de novo clusters.

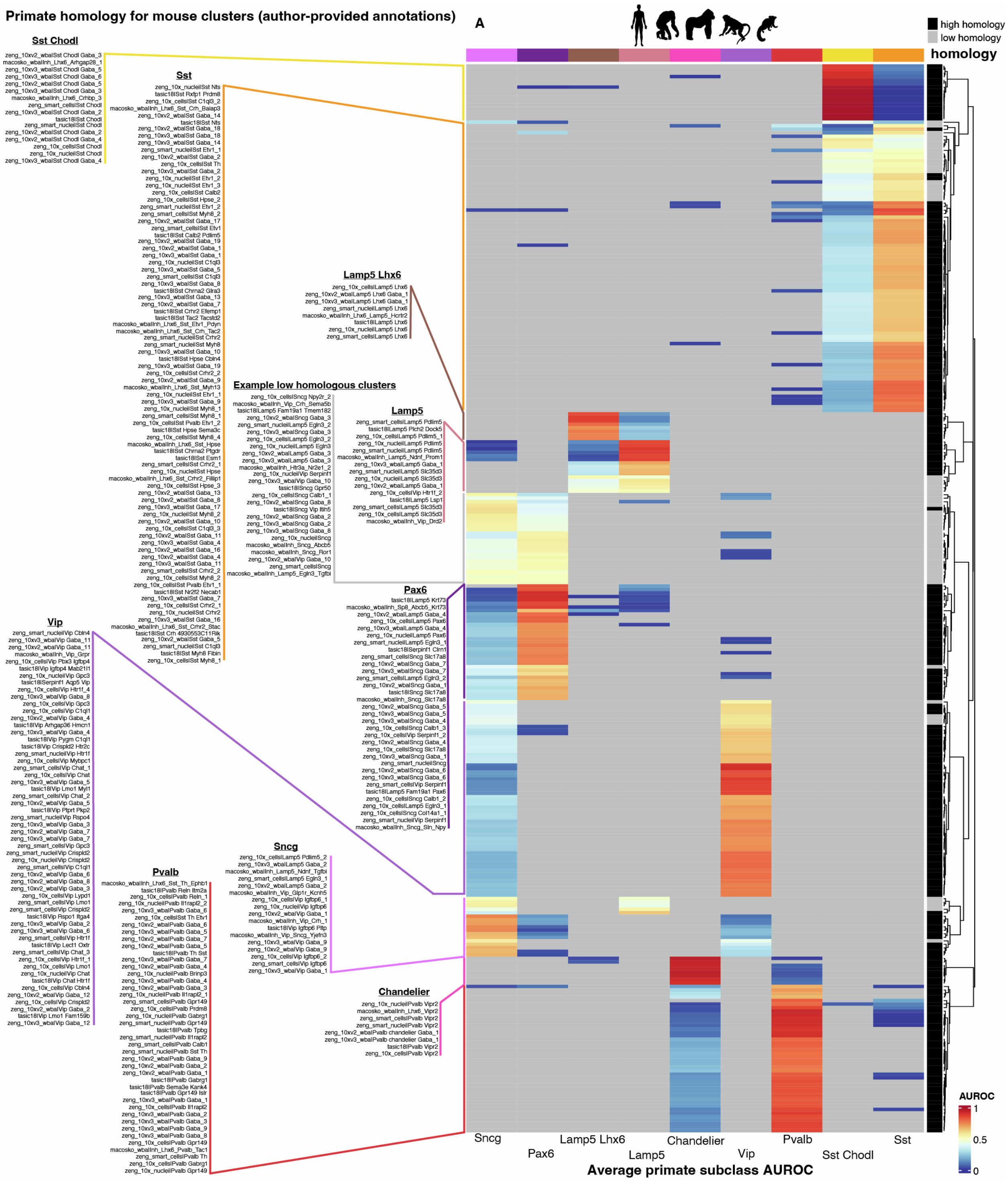

Primate homology for mouse clusters (author-provided annotations)
A
high homology
low homology
homology
Sst Chodl
Sst
Lamp5 Lhx6
Example low homologous clusters
Lamp5
Pax6
Vip
Sncg
Pvalb
Chandelier
AUROC
1
0.5
0
Sncg
Pax6
Lamp5 Lhx6
Lamp5
Chandelier
Vip
Pvalb
Sst Chodl
Sst
Average primate subclass AUROC

Supplemental Figure 6: Average primate subclass MetaNeighbor AUROCs for all replicable mouse clusters

A: Heatmap depicting the average primate subclass one-vs-best MetaNeighbor AUROC (columns) for all of the replicable mouse clusters (rows). The row annotation denotes the mouse clusters with either high (an AUROC >= 0.60) or low (an AUROC < 0.60) homology. The author annotations for all of the replicable mouse clusters are to the left of the heatmap, denoted by their mapped homologous primate subclass annotation. Full annotations are present in Supp. Table 3.

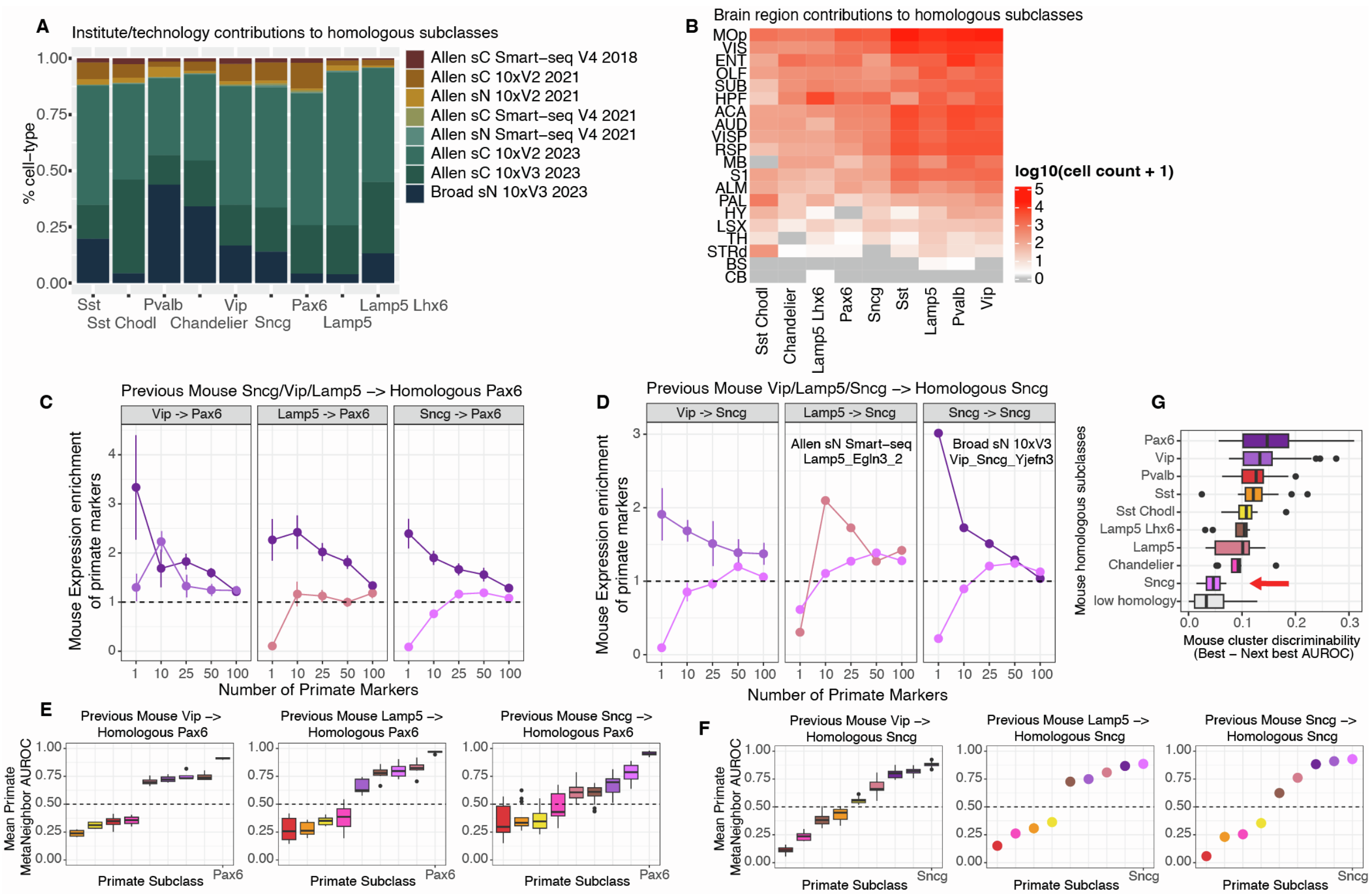
A
Institute/technology contributions to homologous subclasses
% cell−type
Allen sC Smart−seq V4 2018
Allen sC 10xV2 2021
Allen sN 10xV2 2021
Allen sC Smart−seq V4 2021
Allen sN Smart−seq V4 2021
Allen sC 10xV2 2023
Allen sC 10xV3 2023
Broad sN 10xV3 2023
Sst
Sst Chodl
Pvalb
Chandelier
Vip
Sncg
Pax6
Lamp5
Lamp5 Lhx6
B
Brain region contributions to homologous subclasses
MOp
VIS
ENT
OLF
SUB
HPF
ACA
AUD
VISP
RSP
MB
S1
ALM
PAL
HY
LSX
TH
STRd
BS
CB
log10(cell count + 1)
C
Previous Mouse Sncg/Vip/Lamp5 –> Homologous Pax6
Vip -> Pax6
Lamp5 -> Pax6
Sncg -> Pax6
Mouse Expression enrichment of primate markers
Number of Primate Markers
D
Previous Mouse Vip/Lamp5/Sncg –> Homologous Sncg
Vip -> Sncg
Lamp5 -> Sncg
Sncg -> Sncg
Allen sN Smart-seq Lamp5_Egln3_2
Broad sN 10xV3 Vip_Sncg_Yjefn3
G
Mouse homologous subclasses
low homology
Mouse cluster discriminability (Best – Next best AUROC)
E
Previous Mouse Vip –> Homologous Pax6
Previous Mouse Lamp5 –> Homologous Pax6
Previous Mouse Sncg –> Homologous Pax6
Mean Primate MetaNeighbor AUROC
Primate Subclass
F
Previous Mouse Vip –> Homologous Sncg
Previous Mouse Lamp5 –> Homologous Sncg
Previous Mouse Sncg –> Homologous Sncg

Supplemental Figure 7: The Sncg subclass is a weak homologous match between mice and primates

A: Stacked barplot depicting the mouse dataset percentage composition for the 9 homologous mouse subclass annotations.
B: Heatmap depicting the log10(cell count +1) of the 9 homologous mouse subclasses against their regional annotations. ACA: Anterior cingulate area, ALM: Anterior lateral motor cortex, AUD: Auditory areas, BS: Brain stem, CB: Cerebellum, ENT: Entorhinal area, HPF: Hippocampal formation, HY: Hypothalamus, LSX: Lateral septal complex, MD: Midbrain, MOp: Primary motor area, OLF: Olfactory areas, PAL: Pallidum, RSP: Retrosplenial area, S1: Primary somatosensory area, STRd: Striatum dorsal region, SUB: Subiculum, TH: Thalamus, VIS: Visual cortex, VISp: Primary visual cortex.
C: Scatter-line plots comparing the marker set enrichment scores of the markers for the two associated primate subclasses of the mouse clusters that comprise the homologous Pax6 mouse subclass. For example, the left panel depicts the marker enrichment of the primate Vip and Pax6 subclass markers in the mouse clusters that switch from Vip to Pax6 subclass annotations. The points depict the mean marker enrichment score for the mouse clusters, with the lines depicting ± SD.
D: Same as in **C**, but for the mouse clusters that comprise the homologous Sncg subclass annotations. Only a single mouse cluster represents the Lamp5 -> Sncg or the Sncg -> Sncg groups, denoted in the associated panels
E: Boxplots depicting the all-vs-all MetaNeighbor AUROC distributions of all of the primate subclasses for the mouse clusters that comprise the homologous Pax6 subclass annotations.
F: Same as in **E**, but for the mouse clusters that comprise the homologous Sncg subclass annotations.
G: Boxplots depicting the distributions of differences in the best versus next best all-vs-all MetaNeighbor AUROCs comparing the mouse clusters against the primate subclasses, as a measure of discriminability between a mouse clusters best and next best primate subclass match. The grey ‘low homology’ distribution represents the scores for all of the ‘low_homology’ mouse clusters in Supp. Table 3. The red arrow highlights the mouse homologous Sncg subclass as the lowest scoring subclass, next to the low homology clusters, indicating weak replicability between the mouse and primate datasets.

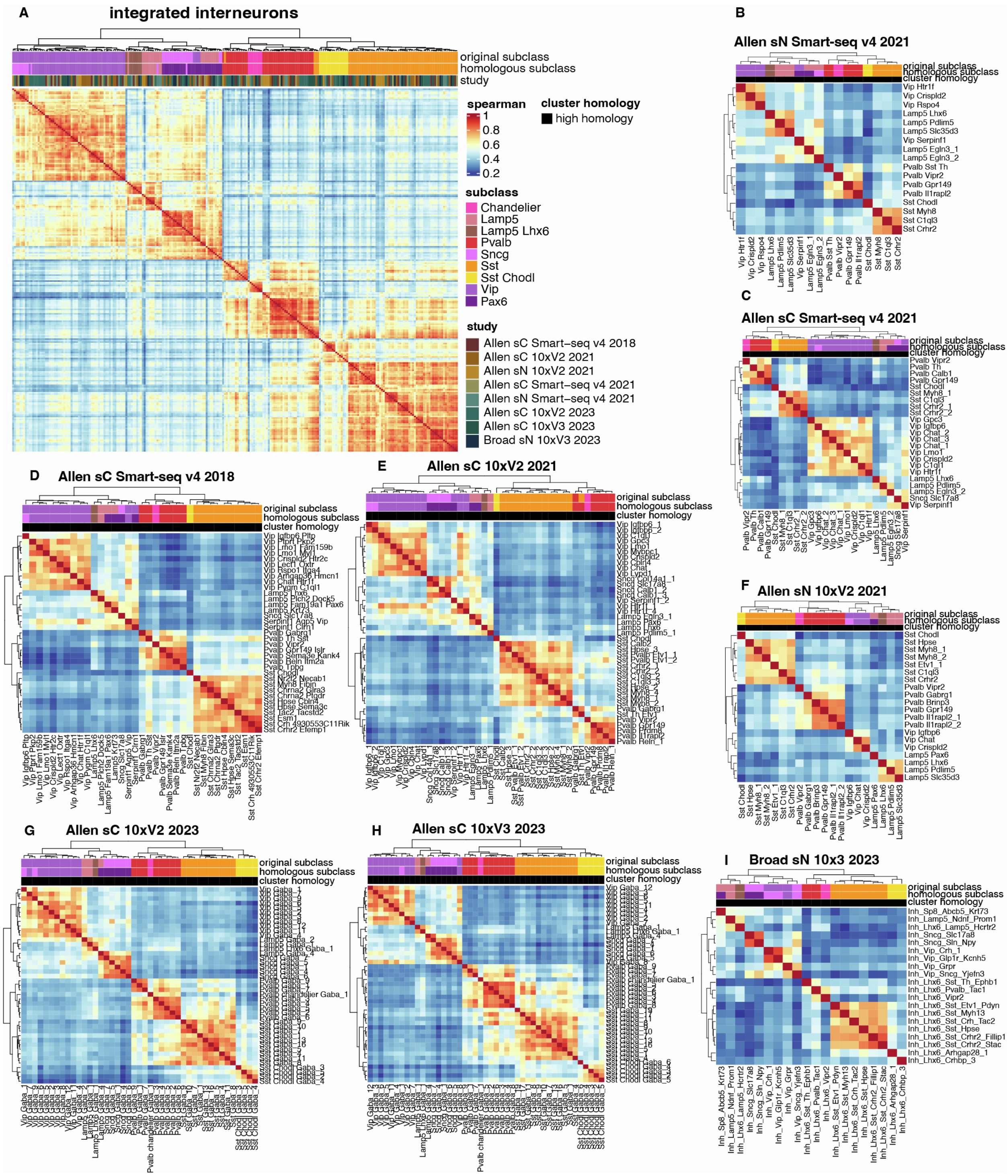
A
integrated interneurons
original subclass
homologous subclass
study
spearman
1
0.8
0.6
0.4
0.2
cluster homology
high homology
subclass
Chandelier
Lamp5
Lamp5 Lhx6
Pvalb
Sncg
Sst
Sst Chodl
Vip
Pax6
study
Allen sC Smart−seq v4 2018
Allen sC 10xV2 2021
Allen sN 10xV2 2021
Allen sC Smart−seq v4 2021
Allen sN Smart−seq v4 2021
Allen sC 10xV2 2023
Allen sC 10xV3 2023
Broad sN 10xV3 2023
B
Allen sN Smart-seq v4 2021
C
Allen sC Smart-seq v4 2021
D
Allen sC Smart-seq v4 2018
E
Allen sC 10xV2 2021
F
Allen sN 10xV2 2021
G
Allen sC 10xV2 2023
H
Allen sC 10xV3 2023
I
Broad sN 10x3 2023
original subclass
homologous subclass
cluster homology

Supplemental Figure 8: Mouse cell-type taxonomies and original vs homologous subclass annotations

A: Cell-type taxonomy (see *Cell-type taxonomies* methods section) of the integrated replicable mouse clusters with high primate homology.
B: Cell-type taxonomy of the Allen sN Smart-seq v4 2021 mouse clusters with high primate homology, using original author annotations.
C: Cell-type taxonomy of the Allen sC Smart-seq v4 2021 mouse clusters with high primate homology, using original author annotations.
D: Cell-type taxonomy of the Allen sC Smart-seq v4 2018 mouse clusters with high primate homology, using original author annotations.
E: Cell-type taxonomy of the Allen sC 10x V2 2021 mouse clusters with high primate homology, using original author annotations.
F: Cell-type taxonomy of the Allen sN 10x V2 2021 mouse clusters with high primate homology, using original author annotations.
G: Cell-type taxonomy of the Allen sC 10x V2 2023 mouse clusters with high primate homology, using original author annotations.
H: Cell-type taxonomy of the Allen sC 10x V3 2023 mouse clusters with high primate homology, using original author annotations.
I: Cell-type taxonomy of the Broad sN 10x V3 2023 mouse clusters with high primate homology, using original author annotations.

sN: single-nucleus, sC: single-cell

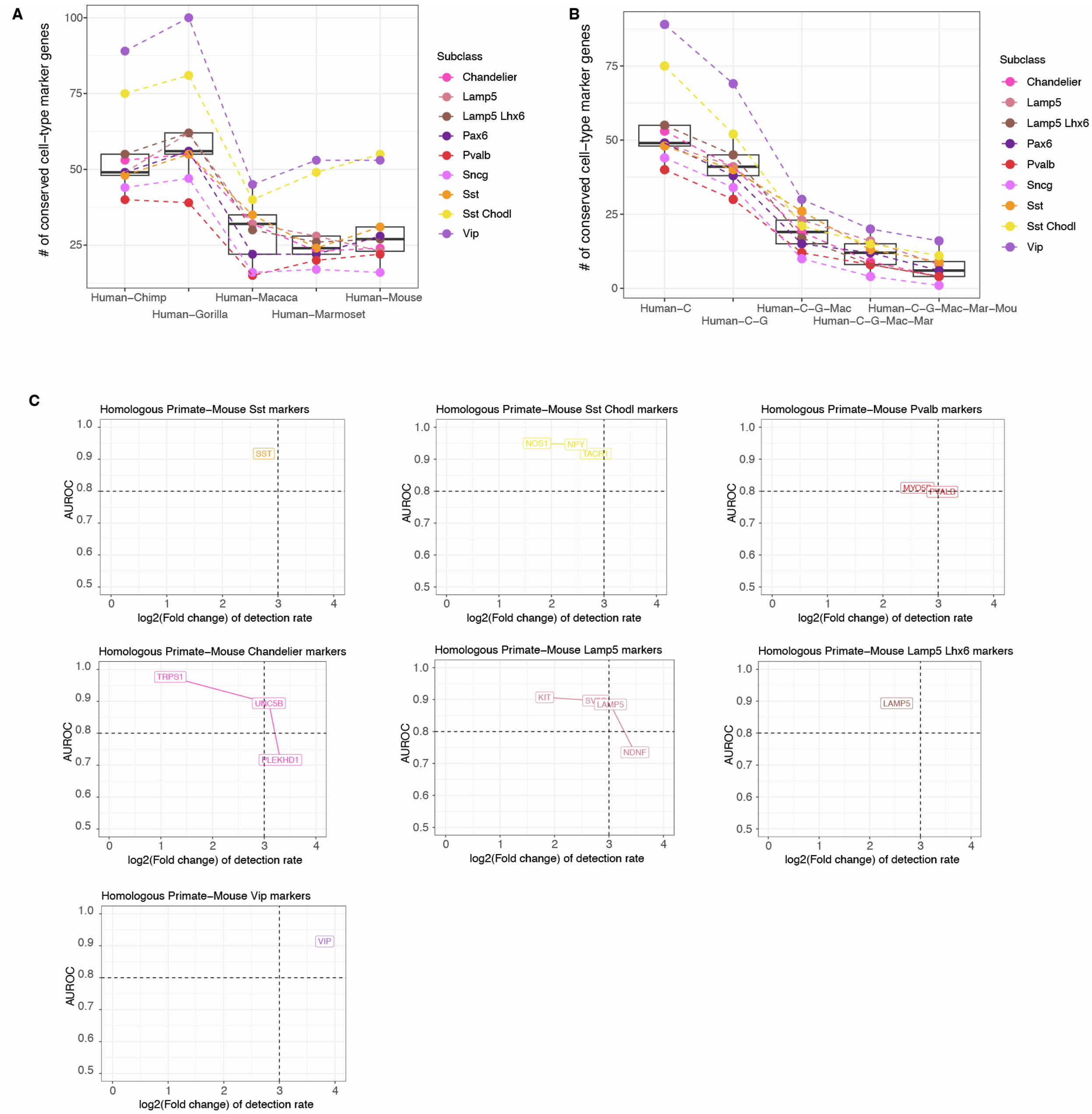
A
# of conserved cell−type marker genes
Human−Chimp
Human−Gorilla
Human−Macaca
Human−Marmoset
Human−Mouse
Subclass
Chandelier
Lamp5
Lamp5 Lhx6
Pax6
Pvalb
Sncg
Sst
Sst Chodl
Vip
B
# of conserved cell−type marker genes
Human−C
Human−C−G
Human−C−G−Mac
Human−C−G−Mac−Mar
Human−C−G−Mac−Mar−Mou
Subclass
Chandelier
Lamp5
Lamp5 Lhx6
Pax6
Pvalb
Sncg
Sst
Sst Chodl
Vip
C
Homologous Primate−Mouse Sst markers
SST
Homologous Primate−Mouse Sst Chodl markers
NOS1
NPY
Homologous Primate−Mouse Pvalb markers
Homologous Primate−Mouse Chandelier markers
TRPS1
Homologous Primate−Mouse Lamp5 markers
KIT
LAMP5
NDNF
Homologous Primate−Mouse Lamp5 Lhx6 markers
LAMP5
Homologous Primate−Mouse Vip markers
VIP
AUROC
log2(Fold change) of detection rate

Supplemental Figure 9: Conservation of top subclass markers across primates and mice

A: Boxplots depicting the number of subclass markers (cross-species log2-fold change >= 4 and an FDR-adjusted p-value <= 0.05) shared between human-species pairs
B: Boxplots depicting the number of subclass markers (cross-species log2-fold change >= 4 and an FDR-adjusted p-value <= 0.05) shared between cumulative species between human and mice.
C: The subclass pareto fronts of the markers conserved across all sampled primate species and mice.

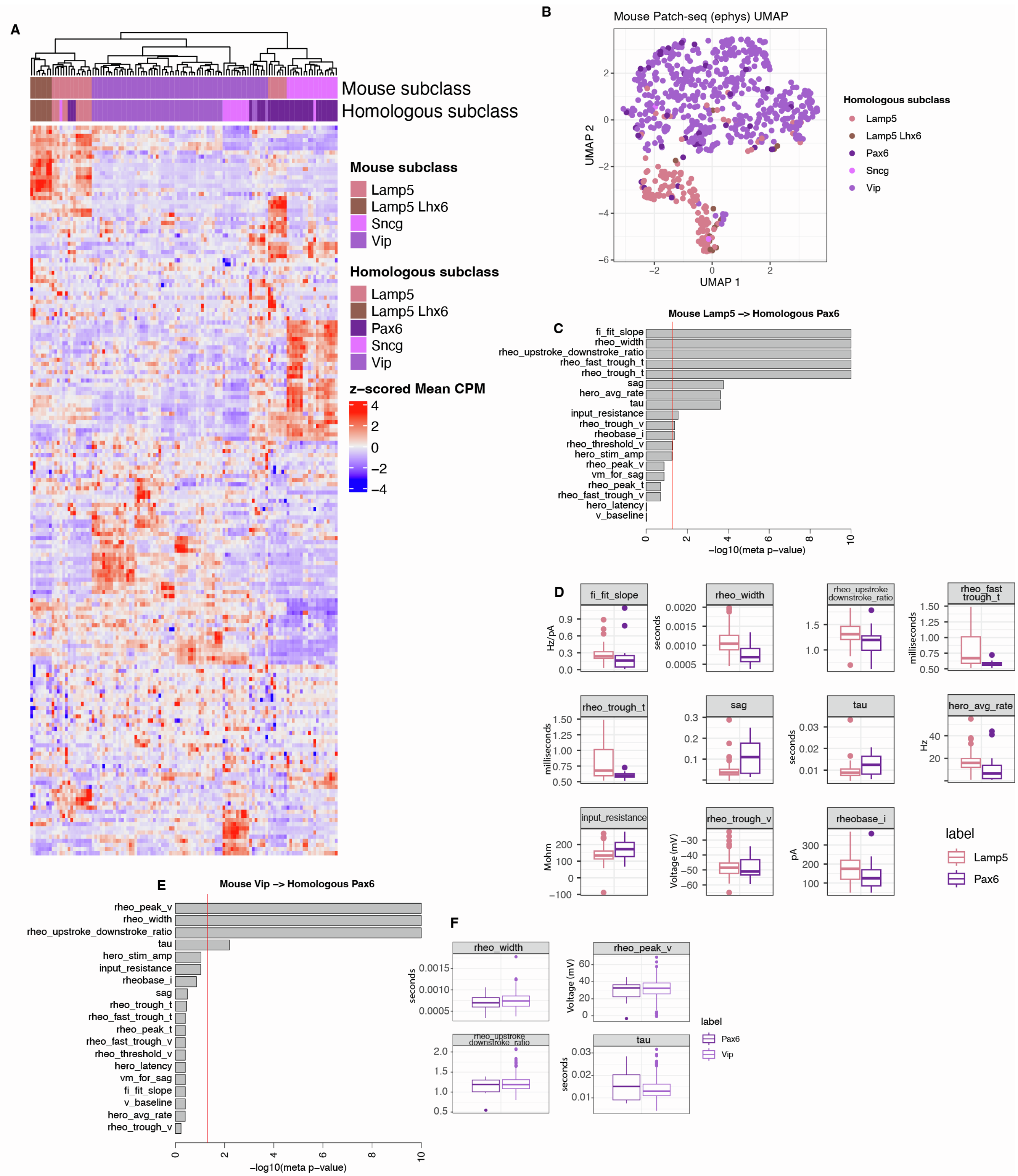
A
Mouse subclass
Homologous subclass
Mouse subclass
Lamp5
Lamp5 Lhx6
Sncg
Vip
Homologous subclass
Lamp5
Lamp5 Lhx6
Pax6
Sncg
Vip
z–scored Mean CPM
4
2
0
−2
−4
B
Mouse Patch-seq (ephys) UMAP
UMAP 2
UMAP 1
Homologous subclass
Lamp5
Lamp5 Lhx6
Pax6
Sncg
Vip
C
Mouse Lamp5 –> Homologous Pax6
fi_fit_slope
rheo_width
rheo_upstroke_downstroke_ratio
rheo_fast_trough_t
rheo_trough_t
sag
hero_avg_rate
tau
input_resistance
rheo_trough_v
rheobase_i
rheo_threshold_v
hero_stim_amp
rheo_peak_v
vm_for_sag
rheo_peak_t
rheo_fast_trough_v
hero_latency
v_baseline
−log10(meta p−value)
D
fi_fit_slope
rheo_width
rheo_upstroke downstroke_ratio
rheo_fast trough_t
rheo_trough_t
sag
tau
hero_avg_rate
input_resistance
rheo_trough_v
rheobase_i
Hz/pA
seconds
milliseconds
Hz
Mohm
Voltage (mV)
pA
label
Lamp5
Pax6
E
Mouse Vip –> Homologous Pax6
rheo_peak_v
rheo_width
rheo_upstroke_downstroke_ratio
tau
hero_stim_amp
input_resistance
rheobase_i
sag
rheo_trough_t
rheo_fast_trough_t
rheo_peak_t
rheo_fast_trough_v
rheo_threshold_v
hero_latency
vm_for_sag
fi_fit_slope
v_baseline
hero_avg_rate
rheo_trough_v
−log10(meta p−value)
F
rheo_width
rheo_peak_v
rheo_upstroke downstroke_ratio
tau
seconds
Voltage (mV)
label
Pax6
Vip

Supplemental Figure 10: Mouse Vip and Pax6 are distinct transcriptionally for ion channel expression, yet similar electrophysiologically

A: Full heatmap depicting the scaled expression of all 176 ion channel genes (Supp. Table 2) across mouse subclasses, whereas Fig. 3B depicts the top 10 differentially expressed genes per original mouse subclass.
B: UMAP of mouse CGE interneurons using electrophysiological features, cells are labeled using the homologous primate subclass annotations.
C: Barplot depicting the aggregate p-values per electrophysiological feature for distinguishing Pax6 cells from their previous Lamp5 annotated subclass. See *Ion channel transcriptomic data and Patch-seq data* methods section.
D: Boxplots comparing the significant electrophysiological features identified in panel G between the Lamp5 cells and the Pax6 cells previously labeled as Lamp5. Additional details on these measurements can be found in the original publication[20].
E: Barplot depicting the aggregate p-value computed per electrophysiological feature for distinguishing Pax6 annotated cells that were previously labeled as Vip from the remaining Vip annotated cells.
F: Boxplots comparing the significant electrophysiological features identified in panel C between the Vip cells and the Pax6 cells previously labeled as Vip.

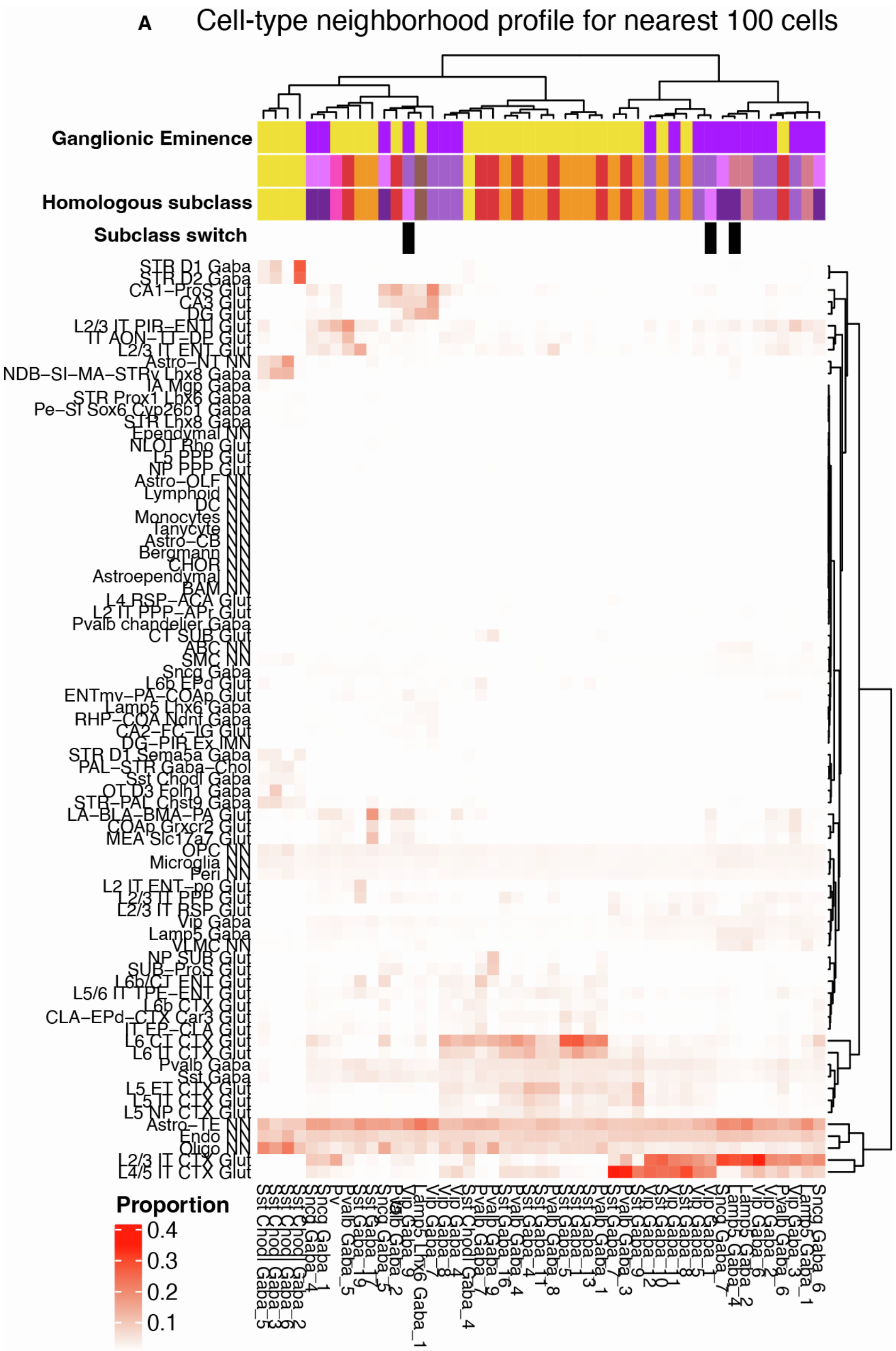
A
Cell-type neighborhood profile for nearest 100 cells
Ganglionic Eminence
Homologous subclass
Subclass switch
STR D1 Gaba
STR D2 Gaba
CA1−ProS Glut
CA3 Glut
DG Glut
L2/3 IT PIR−ENTl Glut
IT AON−TT−DP Glut
L2/3 IT ENT Glut
Astro−NT NN
NDB−SI−MA−STRv Lhx8 Gaba
IA Mgp Gaba
STR Prox1 Lhx6 Gaba
Pe−SI Sox6 Cyp26b1 Gaba
STR Lhx8 Gaba
Ependymal NN
NLOT Rho Glut
L5 PPP Glut
NP PPP Glut
Astro−OLF NN
Lymphoid NN
DC NN
Monocytes NN
Tanycyte NN
Astro−CB NN
Bergmann NN
CHOR NN
Astroependymal NN
BAM NN
L4 RSP−ACA Glut
L2 IT PPP−APr Glut
Pvalb chandelier Gaba
CT SUB Glut
ABC NN
SMC NN
Sncg Gaba
L6b EPd Glut
ENTmv−PA−COAp Glut
Lamp5 Lhx6 Gaba
RHP−COA Ndnf Gaba
CA2−FC−IG Glut
DG−PIR Ex IMN
STR D1 Sema5a Gaba
PAL−STR Gaba−Chol
Sst Chodl Gaba
OT D3 Folh1 Gaba
STR−PAL Chst9 Gaba
LA−BLA−BMA−PA Glut
COAp Grxcr2 Glut
MEA Slc17a7 Glut
OPC NN
Microglia NN
Peri NN
L2 IT ENT−po Glut
L2/3 IT PPP Glut
L2/3 IT RSP Glut
Vip Gaba
Lamp5 Gaba
VLMC NN
NP SUB Glut
SUB−ProS Glut
L6b/CT ENT Glut
L5/6 IT TPE−ENT Glut
L6b CTX Glut
CLA−EPd−CTX Car3 Glut
IT EP−CLA Glut
L6 CT CTX Glut
L6 IT CTX Glut
Pvalb Gaba
Sst Gaba
L5 ET CTX Glut
L5 IT CTX Glut
L5 NP CTX Glut
Astro−TE NN
Endo NN
Oligo NN
L2/3 IT CTX Glut
L4/5 IT CTX Glut
Proportion
0.4
0.3
0.2
0.1

Supplemental Figure 11: Spatial variability of homologous mouse interneuron subclasses at a broad resolution

A: Heatmap depicting the average cell-type neighborhood proportion values for target MGE and CGE clusters (columns), computed using the top 100 nearest neighbors per cell. Columns are at the cluster-level (supertype as defined by ref[16]) and rows are at the subclass-level as defined by ref[16].

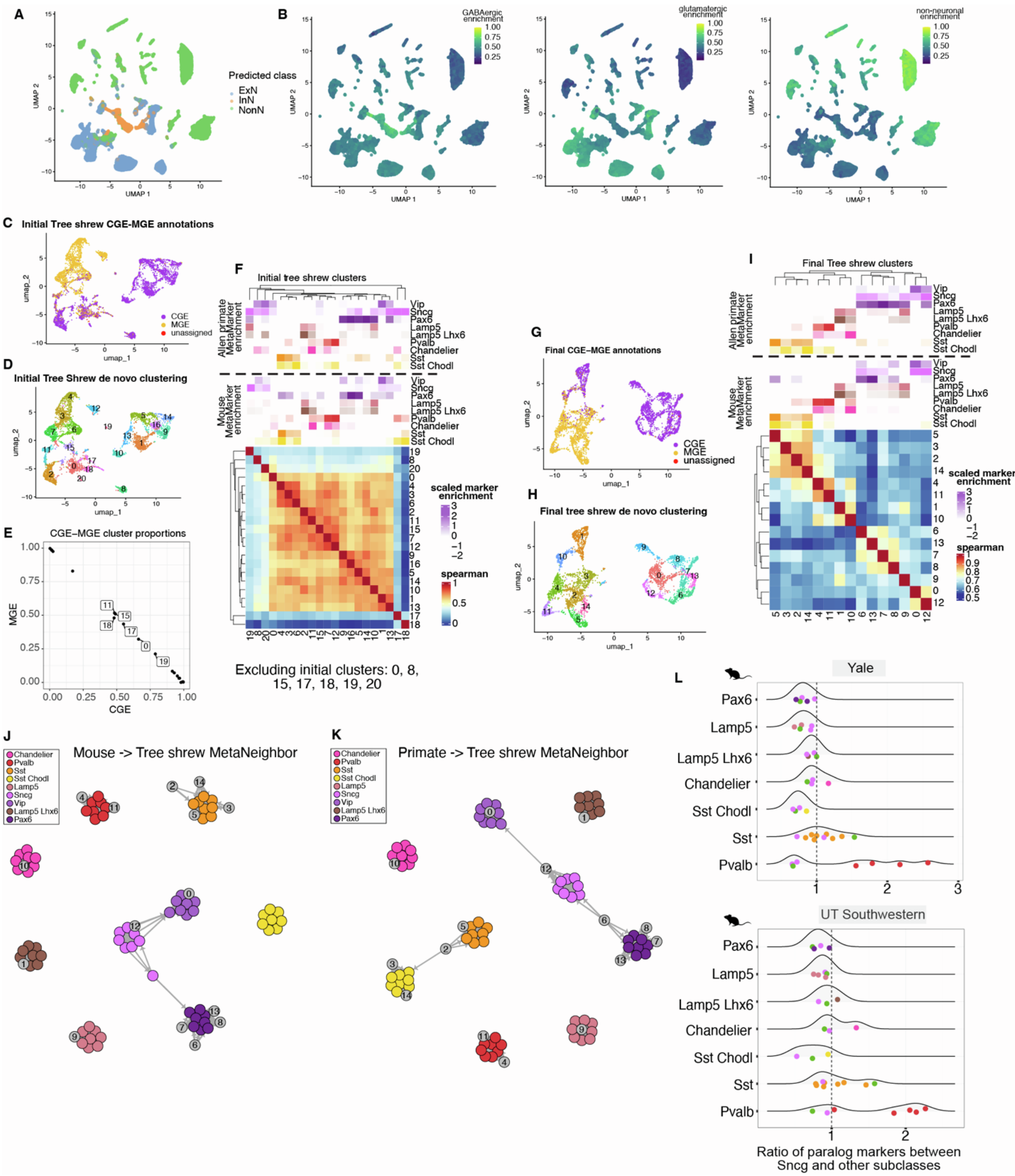
A
Predicted class
ExN
InN
NonN
UMAP 1
UMAP 2
B
GABAergic enrichment
glutamatergic enrichment
non-neuronal enrichment
C Initial Tree shrew CGE-MGE annotations
CGE
MGE
unassigned
D Initial Tree Shrew de novo clustering
E CGE–MGE cluster proportions
MGE
CGE
F Initial tree shrew clusters
Allen primate MetaMarker enrichment
Mouse MetaMarker enrichment
Vip
Sncg
Pax6
Lamp5
Lamp5 Lhx6
Pvalb
Chandelier
Sst
Sst Chodl
scaled marker enrichment
spearman
Excluding initial clusters: 0, 8, 15, 17, 18, 19, 20
G Final CGE–MGE annotations
H Final tree shrew de novo clustering
I Final Tree shrew clusters
J Mouse -> Tree shrew MetaNeighbor
K Primate -> Tree shrew MetaNeighbor
L
Yale
UT Southwestern
Ratio of paralog markers between Sncg and other subclasses

Supplemental Figure 12: Tree-shrew processing and mouse paralogous marker cell-type ratios

A: UMAP depicting the full tree-shrew dataset with the initial Excitatory, Inhibitory, and Non-neuronal annotations (see the *Annotating Tree shrew de novo clusters* methods section)

B: Same UMAP as in **A**, but depicting either the Excitatory, Inhibitory, or Non-neuronal MetaMarker enrichment scores, max-normalized.

C: UMAP depicting the Inhibitory cells annotated as CGE or MGE (see the *Annotating Tree shrew de novo clusters* methods section).

D: Same UMAP as in **C**, but depicting the de novo clusters.

E: Scatter plot comparing the CGE and MGE cluster proportions for the de novo clusters. Clusters with less than 80% CGE or MGE composition are labeled. All of these CGE-MGE intermediate clusters were excluded, with the exception of cluster 11 due to its clear high enrichment of Chandelier marker expression, in panel **F**.

F: Cell-type taxonomy of the tree shrew de novo clusters with the associated mouse or primate subclass MetaMarker expression enrichment scores, as in Figure 5A and E. Clusters 8 and 20 were additionally excluded due to their poor transcriptomic similarity to other clusters and a lack of clear enrichment for any of the subclass labels.

G: UMAP depicting the final CGE and MGE annotations after the cluster filtering

H: Same UMAP as in **G**, but depicting the final set of de novo clusters.

I: Cell-type taxonomy of the final tree-shrew de novo clusters with the associated mouse or primate subclass MetaMarker expression enrichment scores.

J: Graph plot depicting the one-vs-best MetaNeighbor scores between the mouse homologous subclasses and the tree-shrew de novo clusters. Edges denote AUROCs >= 0.60, edge length corresponds inversely to AUROCs (high AUROCs -> small edges).

K: Same as in **G**, but depicting the Allen MTG primate subclasses and the tree-shrew de novo clusters.

L: Density and scatter plots depicting the ratios of paralogous markers for human vs mouse subclass comparisons. Same plot as in Figure 6E for the mouse, but with all the other subclasses other than Vip against the Sncg subclass.

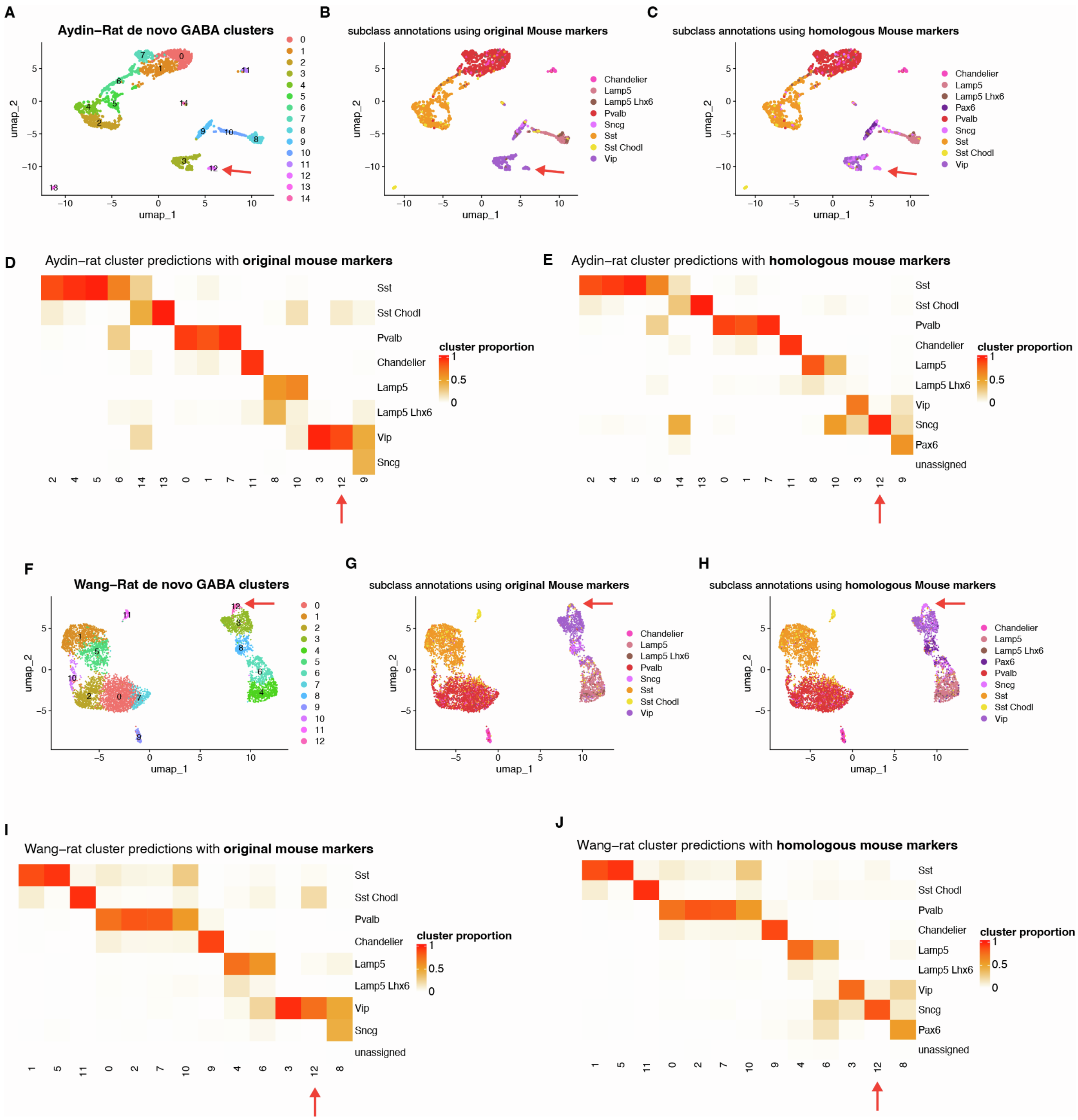

A
Aydin–Rat de novo GABA clusters
umap_1
umap_2
0 1 2 3 4 5 6 7 8 9 10 11 12 13 14
B
subclass annotations using original Mouse markers
Chandelier
Lamp5
Lamp5 Lhx6
Pvalb
Sncg
Sst
Sst Chodl
Vip
C
subclass annotations using homologous Mouse markers
Chandelier
Lamp5
Lamp5 Lhx6
Pax6
Pvalb
Sncg
Sst
Sst Chodl
Vip
D
Aydin-rat cluster predictions with original mouse markers
Sst
Sst Chodl
Pvalb
Chandelier
Lamp5
Lamp5 Lhx6
Vip
Sncg
cluster proportion
1
0.5
0
2 4 5 6 14 13 0 1 7 11 8 10 3 12 9
E
Aydin–rat cluster predictions with homologous mouse markers
Sst
Sst Chodl
Pvalb
Chandelier
Lamp5
Lamp5 Lhx6
Vip
Sncg
Pax6
unassigned
cluster proportion
1
0.5
0
2 4 5 6 14 13 0 1 7 11 8 10 3 12 9
F
Wang–Rat de novo GABA clusters
umap_1
umap_2
0 1 2 3 4 5 6 7 8 9 10 11 12
G
subclass annotations using original Mouse markers
Chandelier
Lamp5
Lamp5 Lhx6
Pvalb
Sncg
Sst
Sst Chodl
Vip
H
subclass annotations using homologous Mouse markers
Chandelier
Lamp5
Lamp5 Lhx6
Pax6
Pvalb
Sncg
Sst
Sst Chodl
Vip
I
Wang–rat cluster predictions with original mouse markers
Sst
Sst Chodl
Pvalb
Chandelier
Lamp5
Lamp5 Lhx6
Vip
Sncg
unassigned
cluster proportion
1
0.5
0
1 5 11 0 2 7 10 9 4 6 3 12 8
J
Wang–rat cluster predictions with homologous mouse markers
Sst
Sst Chodl
Pvalb
Chandelier
Lamp5
Lamp5 Lhx6
Vip
Sncg
Pax6
unassigned
cluster proportion
1
0.5
0
1 5 11 0 2 7 10 9 4 6 3 12 8

Supplemental Figure 13: Subclass annotations of Rat brain data

A: UMAP of the GABAergic de novo clusters for the Aydin 2024 rat cortex dataset
B: Same UMAP as in A, depicting single-cell subclass annotations predicted using the top 50 markers from the original mouse subclass annotations.
C: Same UMAP as in A, depicting single-cell subclass annotations predicted using the top 50 markers from the homologous mouse subclass annotations.
D: Heatmap depicting the Aydin 2024 de novo cluster proportions mapped to mouse subclass annotations using markers derived from the original mouse subclass annotations
E: Heatmap depicting the Aydin 2024 de novo cluster proportions mapped to mouse subclass annotations using markers derived from the homologous mouse subclass annotations.
F: UMAP of the GABAergic de novo clusters for the Wang 2024 rat cortex dataset
G: Same UMAP as in F, depicting single-cell subclass annotations predicted using the top 50 markers from the original mouse subclass annotations.
H: Same UMAP as in F, depicting single-cell subclass annotations predicted using the top 50 markers from the homologous mouse subclass annotations.
I: Heatmap depicting the Wang-Rat de novo cluster proportions mapped to mouse subclass annotations using markers derived from the original mouse subclass annotations
J: Heatmap depicting the Wang 2024 de novo cluster proportions mapped to mouse subclass annotations using markers derived from the homologous mouse subclass annotations.

All red arrows highlight the cluster per dataset that is predominately annotated Vip using original Vip subclass mouse markers and then is predominately annotated Sncg using the homologous Sncg subclass mouse markers. Cluster 12 in both the Aydin and Wang datasets.
